# Pleiotropic Properties of Amphiphilic Dihydropyridines, Dihydropyridones, and Aminovinylcarbonyl Compounds

**DOI:** 10.1155/2020/8413713

**Published:** 2020-12-31

**Authors:** Martins Rucins, Rufus Smits, Anda Sipola, Brigita Vigante, Ilona Domracheva, Baiba Turovska, Ruslan Muhamadejev, Karlis Pajuste, Mara Plotniece, Arkadij Sobolev, Gunars Duburs, Aiva Plotniece

**Affiliations:** ^1^Latvian Institute of Organic Synthesis, Aizkraukles 21, Riga LV-1006, Latvia; ^2^Department of Pharmaceutical Chemistry, Faculty of Pharmacy, Riga Stradiņš University, Dzirciema 16, Riga LV-1007, Latvia

## Abstract

Three groups of synthetic lipids are chosen for studies: (1) 1,4-dihydropyridines (1,4-DHPs) containing two cationic moieties and their analogues; (2) 3,4-dihydro-2(1*H*)-pyridones containing a cationic moiety; and (3) acyclic, open-chain analogues, i.e., 2-amino-3-alkoxycarbonylalkylammonium derivatives. 1,4-DHPs possessing dodecyl alkyl chains in the ester groups in positions 3 and 5 and cationic nitrogen-containing groups in positions 2 and 6 have high cytotoxicity in cancer cells HT-1080 (human lung fibrosarcoma) and MH-22A (mouse hepatoma), but low cytotoxicity in the noncancerous NIH3T3 cells (mouse embryonic fibroblast). On the contrary, similar compounds having short (methyl, ethyl, or propoxyethyl) chains in the ester groups in positions 3 and 5 lack cytotoxicity in the cancer cells HT-1080 and MH-22A even at high doses. Inclusion of fluorine atoms in the alkyl chains in positions 3 and 5 of the DHP cycle decreases the cytotoxicity of the mentioned compounds. Structurally related dihydropyridones with a polar head group are substantially more toxic to normal and cancerous cells than the DHP analogues. Open-chain analogues of DHP lipids comprise the same conjugated aminovinylcarbonyl moiety and possess anticancer activity, but they also have high basal cytotoxicity. Electrochemical oxidation data demonstrate that oxidation potentials of selected compounds are in the range of 1.6–1.7 V for cationic 1,4-DHP, 2.0–2.4 V for cationic 3,4-dihydropyridones, and 1.2–1.5 V for 2-amino-3-alkoxycarbonylalkylammonium derivatives. Furthermore, the tested cationic 1,4-DHP amphiphiles possess antiradical activity. Molecular topological polar surface area values for the tested compounds were defined in accordance with the main fragments of compound structures. The determined log*P* values were highest for dodecyl ester groups in positions 3 and 5 of the 1,4-DHP and lowest for short alkyl chain-containing amphiphiles.

## 1. Introduction

For a long time, liposomes have been considered to have a role in the encapsulation of toxic drugs (mainly anticancer drugs) into liposomal drug delivery systems which are supposed to be inert, having no pharmacological or chemotherapeutical activity *per se* [1]. The properties of liposomes are mainly dependent on the characteristics of their constituent lipids.

Lipids and liposomal agents as liposome-forming building blocks depending on their physicochemical properties can influence the immune system. For the development of new liposomal systems, it is important to perform analysis on the liposome-forming lipid properties with an emphasis on toxicity to healthy tissues and immune responses [2]. Synthetic cationic amphiphilic bilayers can act as gene, vaccine, or drug carriers; at the same time, they can interact with negatively charged prokaryotic or eukaryotic cells, causing cell adhesion and loss of cell viability. Lipids and liposomes deserve to be investigated as potential drugs [3]. Cytotoxicity of cationic liposomes is a significant property to be checked [4].

Liposomes may induce oxidative damage to normal tissues [5]. Reactive oxygen species (ROS) and lipid peroxidation products not only are cytotoxic but may also perform and modulate signal transduction in cells [6]. It was demonstrated that the levels of ROS and the activity of scavenging/antioxidant enzymes in drug-resistant cancer cells are typically increased compared to nonresistant cancer and normal cells. Thereby, multidrug-resistant (MDR) cancer cells may be more susceptible to alterations in ROS levels. Numerous studies suggest that compounds modulating cellular ROS levels can enhance MDR cancer cell death and sensitize MDR cancer cells to certain chemotherapeutic drugs [7]. It was concluded that unspecific elimination of ROS by use of low molecular mass antioxidants was not successful for disease initiation and progression. However, controlling specific ROS-mediated signaling pathways by selective targeting offers a perspective for more refined redox medicine in the future [8]. Additional antioxidant activity (antiradical activity) could be beneficial as it would protect cells and organisms in case of oxidative stress or, in general, be involved in the process of redox regulation and master switch systems [9]. In this way, 1,4-dihydropyridines, being a group of synthetic antioxidants, could be used for the modulation of cellular redox signaling. Lipids reveal cancer cell-selective cytotoxicities—they are less cytotoxic in noncancerous healthy cells [10]. Evaluation of the cytotoxicity of nanoparticles and their components is crucial for the accurate interpretation of pharmacological activities [11]. Nonviral synthetic lipid vectors may interact with biomolecules through functional moieties (biosignature), resulting in biological impacts: intrinsic genomic and nongenomic effects [12].

Our research group has developed liposome-forming cationic 1,4-dihydropyridine (1,4-DHP) amphiphiles capable of transfecting pDNA into different cell lines *in vitro*. To assess the influence of different molecular architectures on gene delivery properties, numerous 1,4-DHP amphiphiles were studied [13–15]. Besides, some of these amphiphilic compounds revealed anticancer activity in HT-1080 and MH-22A cells and antiradical activities (27–40% in DPPH tests) [13, 16]. The remarkable increase of N-H acidity (up to pKa ~7–8) in the DHP cycle is the basis for its buffering activity in these types of gene transfection agents [17].

Recently, it was demonstrated that 4-(N-alkylpyridinium)-1,4-dihydropyridines possess toxicity in Gram-positive and Gram-negative bacteria species and eukaryotic microorganisms [18]. The abovementioned 4-(N-alkylpyridinium)-1,4-dihydropyridines also possess calcium channel-blocking and antioxidant activities [19].

In this work, we chose amphiphilic dihydropyridine derivatives as synthetic lipids and their structural analogues as objects to study pleiotropic activities. The cytotoxic properties of 3 types of amphiphilic compounds in 3 cell lines, namely, normal NIH3T3 (mouse embryonic fibroblast), cancerous HT-1080 (human lung fibrosarcoma), and MH-22A (mouse hepatoma), were evaluated. The studied compounds could be divided into 3 groups according to their structural fragments: (a) 1,4-dihydropyridines containing two cationic moieties as a conjugated cyclic bis(*β*-carbonylvinyl)amino system and some structurally related compounds; (b) 3,4-dihydro-2(1*H*)-pyridones containing a cationic moiety as an isomeric 3,4-dihydropyridine structure with an additional intracyclic carbonyl group—a N-*β*-carbonylvinylamido system; and (c) 2-amino-3-alkoxycarbonylalkylammonium derivatives as acyclic, open-chain *β*-aminovinylcarbonyl systems. Lipophilicity of the selected compounds was characterized using log*P* and molecular topological polar surface area calculations. Evaluation of electrochemical oxidation potentials of the selected compounds was also performed.

## 2. Materials and Methods

### 2.1. Chemistry

More detailed descriptions of synthetic procedures and characterization of the original unpublished intermediates and compounds are described in the Supplementary data.

#### 2.1.1. Synthesis of Cationic 1,4-Dihydropyridines **1–26**

Briefly, the elaborated synthesis of the cationic 1,4-DHP **1**–**7**, **9**–**25** involved three sequential steps. The first step was the synthesis of the corresponding 2,6-dimethyl 1,4-DHP derivative in a two-component Hantzsch-type cyclization using 1.0 eq of the corresponding aldehyde, 1.2 eq of an ammonia source, and 2.0 eq of the corresponding acetoacetate for symmetrically substituted 1,4-DHP derivatives or 1 eq of the corresponding acetoacetate and 1.0 eq of the corresponding 3-aminobut-2-enoate for unsymmetrically substituted ones. The second step involved the bromination of the methyl groups of the 2,6-dimethyl-1,4-DHP derivative with N-bromosuccinimide, and the third step was the nucleophilic substitution of bromine of the 2,6-dibromomethylene-1,4-DHP by N-heterocycles or amines yielding the target compounds. The first step for the synthesis of the parent 1,4-dihydropyridine in the case of compound **26** involved the reaction between 1.0 eq of the corresponding aldehyde, 2.4 eq of ammonium acetate, and 4.0 eq of the corresponding acetoacetate. More detailed synthetic procedures and characterization of the original compounds are described in the Supplementary data.

#### 2.1.2. Synthesis of Compound **8** [20]

Briefly, the condensation of ethyl 4-chloroacetoacetate with glyoxylic acid monohydrate in the presence of piperidine/acetate provided (*E,Z*)-2-(2-chloroacetyl)-but-2-enedioic acid 1-ethyl ester, which was used in the next reaction with ethyl 3-amino-4-chlorobut-2-enoate. The obtained 3,5-diethyl 2,6-bis(chloromethyl)-1,4-dihydropyridine-3,4,5-tricarboxylate was esterified with methanol to afford the parent 2,6-dichloromethylene-1,4-DHP. The target compound **8** was obtained *via* nucleophilic substitution of chlorine by pyridine in the presence of potassium iodide.

#### 2.1.3. Synthesis of Cationic Pyridine **27** [14]

Briefly, the corresponding 2,6-dibromomethylene-1,4-DHP was oxidized by HNO_2_ followed by nucleophilic substitution of bromine with pyridine to give the target compound **27**.

#### 2.1.4. Synthesis of Cationic 3,4-Dihydro-2(1*H*)-pyridones **28**–**38**

Briefly, 3,4-dihydro-2(1*H*)-pyridone synthesis employed a four-component reaction using Meldrum's acid by a heterocyclization with the corresponding *β*-ketoester and the corresponding aldehyde. The obtained 3,4-dihydro-2(1*H*)-pyridones were treated with bromine giving the respective 6-methylbromides, which in subsequent reaction with pyridine or amine provided the target compounds **28**–**38**.

#### 2.1.5. Synthesis of 2-Amino-3-alkoxycarbonylalkylammonium Halides **39**–**48**

Briefly, the first step included the transesterification of the commercially available ethyl 4-chloroacetoacetate with a corresponding carbinol without solvent by azeotropic removal of ethanol. The further reaction of the corresponding 4-chloro-3-oxobutanoates with ammonium acetate led to enaminoesters, which were used for quaternization by tertiary amines or heterocycles giving the target compounds **39**–**48**. Potassium iodide or sodium bromide additives were used for obtaining the corresponding iodides or bromides.

### 2.2. Experiments for Evaluation of Electrochemical Oxidation Potentials

Cyclic voltammetry experiments were carried out on a PARSTAT 2273 electrochemical system. A stationary glassy carbon disk electrode (*d* = 0.5 mm) served as the working electrode, while the counterelectrode was a Pt wire. The oxidation potentials were measured relative to a Ag/Ag^+^ reference electrode. Acetonitrile was dried over P_2_O_5_ and distilled; the distillate was stored over CaH_2_ and redistilled just before use. Recrystallized tetrabutylammonium tetrafluoroborate (TBABF_4_) was used as a supporting electrolyte at 0.1 M concentration.

### 2.3. Determination of Log*P* and Molecular Topological Polar Surface Area

Log*P* and Topological Polar Surface Area (TPSA) values were calculated with the Chem3D Ultra 19 program (PerkinElmer Informatics). For log*P* calculations, the Molecular Networks module was used on single-molecule compounds (salts were taken as cations).

### 2.4. Cytotoxicity

Cytotoxicity of the compounds was determined on HT-1080 (human lung fibrosarcoma) and MH-22A (mouse hepatoma) cell lines and on the normal NIH3T3 (mouse embryonic fibroblast) cell line. IC_50_ is the compound concentration (*μ*g/ml) at which 50% of the cells die. CV is a triarylmethane dye that can bind to ribose-type molecules such as DNA in nuclei. CV staining can be used to quantify the total DNA of the remaining population and thus is used to determine the number of live cells based on the concentration of the dye which remains after staining. MTT is a standard colorimetric assay used to measure cellular proliferation. Yellow MTT (3-(4,5-dimethylthiazol-2-yl)-2,5-diphenyltetrazolium bromide) is reduced to purple formazan in the mitochondria of living cells.

Reduction takes place only when mitochondrial reductase enzymes are active, and therefore, conversion is directly related to the number of viable cells which can be quantified by the absorbance of the solution (between *λ* = 500 and 600 nm) using a spectrophotometer.

### 2.5. Cell Culture and Measurement of Cell Viability

Tumor cell lines HT-1080 (human connective tissue fibrosarcoma, ATCC® CCL-121™) and MH-22A (mouse hepatocarcinoma, ECACC, cat. Nr. 96121721) were used.

HT-1080 and MH-22A cells were seeded in 96-well plates in Dulbecco's modified Eagle's (DMEM) medium containing 10% fetal bovine serum and 4 mM L-glutamine, without antibiotics, and cultivated for 72 h by exposure to different concentrations of compounds. Cell viability was measured using 3-(4,5-dimethylthiazol-2-yl)-2,5-diphenyltetrazolinium bromide (MTT). In brief, after incubating with compounds, the culture medium was removed and fresh medium with 0.2 mg/ml MTT was added in each well of the plate. After incubation (3 h, 37°C, 5% CO_2_), the medium with MTT was removed, and 200 *μ*l DMSO was added at once to each sample. The samples were tested at 540 nm on a Tecan Infinite M1000 multiplate reader. The IC_50_ was calculated using the program GraphPad Prism® 3.0.

For the CV assay, cells were stained with 0.05% crystal violet (Sigma-Aldrich) in 30% methanol for 20 minutes at room temperature. After incubation, the staining solution was removed. The cells were washed 4 times with water. For dye solubilization, 200 *μ*l of a solubilizing solution (0.1 M citrate buffer, pH 4.2 in 50% ethanol; 1 : 1 *v*/*v*) was added. The absorbance of the solution was measured using a Tecan Infinite M1000 multiplate spectrophotometer at a wavelength of 570 nm [21].

### 2.6. Basal Cytotoxicity Test

The Neutral Red Uptake (NRU) assay was performed according to the standard protocol of Stokes et al. [22] modified by a NICEATM-ECVAM validation study [23]. The NRU cytotoxicity assay procedure is based on the ability of viable cells to incorporate and bind neutral red, a supravital dye.

Balb/c NIH 3T3 (mouse Swiss albino embryo fibroblast, ATCC® CRL-1658™) cells (9000 cells/well) were placed into 96-well plates for 24 h in Dulbecco's modified Eagle's medium (DMEM) containing 5% fetal bovine serum. Then, the cells were exposed to the test compound over a range of seven concentrations (1000, 316, 100, 31, 10, 3, and 1 *μ*g/ml) for 24 h. Untreated cells were used as a control. After 24 h, the medium was removed from all plates. Then, 150 *μ*l of neutral red solution was added (0.05 mg/ml NR in DMEM 24 h preincubated at 37°C and then filtered before use through a 0.22 *μ*m syringe filter). Plates were incubated for 3 h, and then, the cells were washed three times with PBS. The dye within viable cells was released by extraction with a mixture of acetic acid, ethanol, and water (1 : 50 : 49). The absorbance of neutral red was measured using a spectrophotometer multiplate reader (Tecan Infinite M1000) at 540 nm. The optical density (OD) was calculated using the following formula: OD (treated cells) × 100/OD (control cells). The IC_50_ values were calculated using the GraphPad Prism® 3.0 program.

### 2.7. Estimation of LD_50_ from IC_50_ Values

Data from the *in vitro* tests were used for estimating the starting dose for acute oral systemic toxicity tests in the rodent. The *in vivo* starting dose is an estimated LD_50_ value calculated by inserting the *in vitro*IC_50_ value into a regression formula: log LD_50_ (mM/kg) = 0.439 log IC_50_ (mM) + 0.621 [23–25]. The value is recalculated to mg/kg, and compounds are evaluated in accordance with 4 toxicity categories [26]: category 1—LD_50_ ≤ 5 mg/kg (highly toxic); category 2—5 < LD_50_ ≤ 50 mg/kg (moderately toxic); category 3—50 < LD_50_ ≤ 300 mg/kg (slightly toxic); and category 4—300 < LD_50_ ≤ 2000 mg/kg (practically nontoxic). Using an alternative *in vitro* method allows the comparison between possibly toxic new compounds and selecting compounds for further study vastly reducing the number of animal experiments.

## 3. Results and Discussion

### 3.1. Structures of Compounds

The studied compounds could be divided into 3 groups considering structure fragments:

1. *1st group*: twenty-five representatives of two cationic moieties containing 1,4-dihydropyridines (compounds **1**–**25** in Table 1) and, additionally, four cationic moieties containing 1,4-dihydropyridine (compound **26** in Table 1) and one as an oxidized form—two cationic moieties containing pyridine (compound **27** in Table 1)
2. *2nd group*: eleven representatives of 3,4-dihydro-2(1*H*)-pyridones containing a cationic moiety as an isomeric 3,4-dihydropyridine structure with an additional intracyclic carbonyl group, i.e., the N-*β*-carbonylvinylamido system (compounds **28**–**38** in Table 2)
3. *3rd group*: ten representatives of cationic 2-amino-3-alkoxycarbonylalkylammonium derivatives as acyclic, open-chain analogues of cyclic 1,4-DHPs (compounds **39**–**48** in Table 3)

These three groups were selected after analysis of their structure-activity relationships as synthetic lipid-like amphiphilic compounds. Previously, the 3rd group representatives—(2-amino-3-alkoxycarbonylalkyl)trialkylammonium halides—were synthesized, and their ribonucleic acid (RNS) transfection activity was demonstrated [27]. This group covers synthetic lipid-like compounds on the basis of a *β*-aminovinylcarbonyl (AVC) moiety: a conjugated pentade system connected with a lipophilic carbon atom chain and a cationic part (alkylammonium or pyridinium type). These compounds are proposed as an open-chain system compared to the cationic 1,4-DHP or pyridone heterocyclic systems. Structurally, the 2nd group molecules—the cationic pyridone derivatives—are heterocycles which comprise a cyclic AVC system and an additional intracyclic carbonyl group and an extracyclic cation. Previously, the 1st group representatives—1,4-DHP derivatives containing pyridinium moieties—were presented as promising tools for delivery of DNA into target cells [13, 14, 20, 28]. It is noteworthy that, due to cross-conjugation of two AVC systems of the 1,4-DHP cycle, its N-H group is influenced by two electron-withdrawing vinylcarbonyls.

### 3.2. Synthesis of the Amphiphiles

**Scheme 1:**
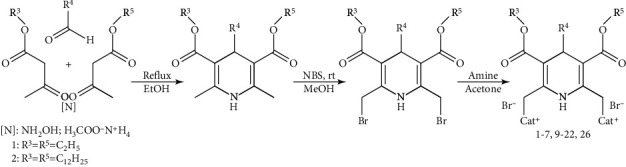
Synthesis of 1,4-dihydropyridine (1,4-DHP) amphiphiles **1**–**7**, **9**–**22**, and **26**.

**Scheme 2:**
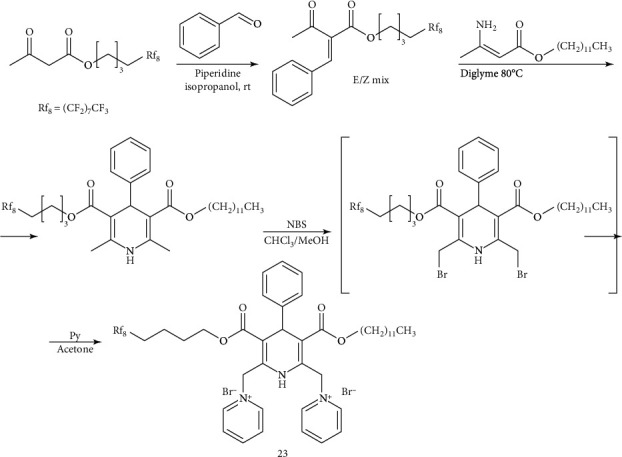
Synthesis of unsymmetrical 1,4-DHP amphiphile **23**.

**Scheme 3:**
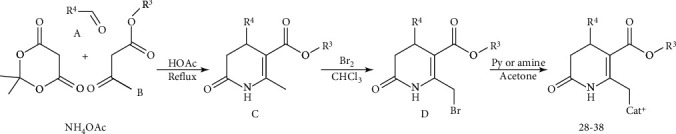
Synthesis of 3,4-dihydro-2(1*H*)-pyridone (DHPDO) amphiphiles **28-38**.

**Scheme 4:**
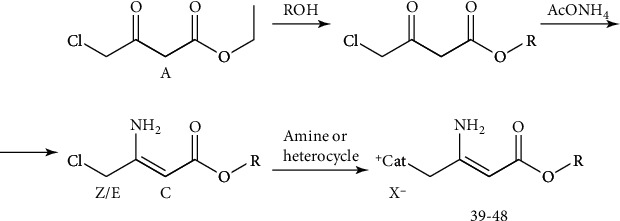
Synthesis of 2-amino-3-alkoxycarbonylalkylammonium halides **39-48**.

Synthesis of the amphiphiles was performed according to Schemes 1–4.

Previously, 3,5-bis(dodecyloxycarbonyl)-1,4-DHPs containing cationic moieties were elaborated as synthetic lipid-like compounds having promising gene delivery properties for DNA transfection; the basic structure-activity relationships have been verified for the cationic 1,4-DHP derivatives as gene delivery systems, and it has been shown that the molecular structure affected their self-assembling properties, pDNA-binding ability, and properties of the formed 1,4-DHP amphiphile-pDNA complexes [13, 15, 28]. The synthetic procedure for the amphiphilic compounds **1**–**7**, **9**–**22**, and **26** is displayed in Scheme 1*via* a multistep sequence. The first step was a Hantzsch synthesis of the parent compound 3,5-bis(alkoxycarbonyl)-2,6-dimethyl-4-phenyl-1,4-dihydropyridine. The second step was bromination of the 2,6-methyl groups with N-bromosuccinimide (NBS) [34] resulting in the second parent compound 2,6-di(bromomethyl)-3,5-bis(alkoxycarbonyl)-4-phenyl-1,4-dihydropyridine. The final step was nucleophilic substitution of bromine in the 2,6-di(bromomethyl)-1,4-DHP by N-heterocycles or amines, which is facile and generally takes place in good yields.

Additionally, for studies of targeted changes in the structure of corresponding dicationic compound **10**, which was found to be more active for DNA delivery among the tested 1,4-DHP amphiphiles [13, 15, 28], 1,1′,1^″^,1‴-((1,4-phenylenebis(3,5-bis((dodecyloxy)carbonyl)-1,4-dihydropyridine-4,2,6-triyl))tetrakis(methylene))tetrakis(pyridin-1-ium) tetrabromide (**26**) was synthesized as a double or a “dimeric” molecule of compound **10** and 1,1′-((3,5-bis(dodecyloxycarbonyl)-4-phenylpyridine-2,6-diyl)bis(methylene))-bis(pyridin-1-ium)dibromide (**27**) was prepared in accordance with what was reported in [14] as the oxidized form of compound **10**. For evaluation of the anion influence on cytotoxicity, 1,1′-((3,5-bis((dodecyloxy)carbonyl)-4-phenyl-1,4-dihydropyridine-2,6-diyl)bis(methylene))bis(pyridin-1-ium) ditetrafluoroborate (**11**) was synthesized from compound **10** after treating with NH_4_BF_4_.

The unsymmetrical 1,4-DHP amphiphile **23** was designed for analysis of the influence of mixed perfluorinated and alkyl ester moiety substituents on the properties of amphiphiles. The 1,4-DHP **23** molecule contains only one of the two esters as a perfluorinated ester moiety while the other is an alkyl ester—the same as previous compounds. The synthetic procedure (Scheme 2) includes a reaction between an enamine and benzylidene in diglyme with inclusion of n-butylpyridinium chloride as a phase transfer catalyst (PTC) [35] to affording the parent dodecyl 5,5,6,6,7,7,8,8,9,9,10,10,11,11,12,12,12-heptadecafluorododecyl 1,4-dihydro-2,6-dimethyl-4-phenylpyridine-3,5-dicarboxylate in 43% yield. Without the PTC, the yield was only 25%. Subsequent bromination with NBS formed the corresponding 2,6-dibromomethylene DHP which was used without further purification in nucleophilic substitution with pyridine yielding the unsymmetrical 1,4-DHP **23**.

The 3,4-dihydro-2(1*H*)-pyridones (DHPDO) possess various pharmacological properties as *α*1a adrenergic receptor antagonists [36], Rho-kinase inhibitors [37], P_2_X_7_ receptor antagonists [38], or G-protein-coupled kinase receptor antagonists [39]. Synthesis of 3,4-dihydro-2(1*H*)-pyridone amphiphiles **28**–**38** was performed according to Scheme 3. In this case, Meldrum's acid was used as the second dicarbonyl component in a Hantzsch-like reaction with heterocyclization, with a corresponding *β*-ketoester and a corresponding aldehyde in the presence of ammonium acetate in refluxing glacial acetic acid [40]. The parent DHPDO solution in chloroform was treated with bromine affording the respective 6-bromomethyl-DHPDO, which on subsequent reaction with pyridine or N,N-dimethyldodecyl-1-amine in dry acetone yielded the corresponding amphiphilic DHPDO derivatives **28**–**38**.

Typically, enaminones have been studied and used as precursors and synthons for the synthesis of novel heterocyclic systems: pyridines, pyrroles, pyrimidines, dihydropyridines, etc. [41]. Therefore, up to now, most of the research in the field of enamines have been devoted to the elaboration of new strategies and synthetic methods, but lack biological studies [42]. 2-Amino-3-alkoxycarbonylalkylammonium halides with long alkyl chains have appeared as a new class of enaminoesters and were elaborated as a transfection agent for RNS transfection [27]. Also, structure analogues—alkyl acyl carnitine esters—were synthesized and characterized as biocompatible cationic lipids for use in gene delivery [43]. Cationic enaminoesters are stable solid compounds with remarkable solubility in water. The synthetic procedure for 2-amino-3-alkoxycarbonylalkylammonium halides **39**–**48** is provided in Scheme 4. Briefly, the first step included the transesterification of commercially available ethyl 4-chloroacetoacetate with the corresponding carbinol without solvent by azeotropic removal of ethanol. Further reaction of the corresponding 4-chloro-3-oxobutanoates with ammonium acetate led to the formation of enaminoesters, which were used for quaternization with tertiary amines or heterocycles by extended heating in a dry solvent. Potassium iodide or sodium bromide additives were used for obtaining the corresponding iodides or bromides.

The perchlorates of amphiphiles **9**, **15**, **22**, **27**, and **32** were obtained from the corresponding bromides by treating with excess of conc. HClO_4_ according to the procedure elaborated by Turovska et al. [44]. In some cases, the perchlorates were used for obtaining solid salts while the corresponding bromides existed as oils, and it was not possible to isolate them from the reaction mixture.

The full description of the synthesis and characterization of the original compounds in detail are given in the Supplementary data. Purity of the studied compounds was at least 97% according to high-performance liquid chromatography (HPLC) data.

### 3.3. Estimation of LD_50_ from IC_50_ Values

It has been proposed that the equation from the correlation of IC_50_ (the concentration of a substance that causes 50% toxicity *in vitro*) could be applied to estimate unknown LD_50_ values for a new compound from IC_50_ values measured as basal cytotoxicity *in vitro*. This estimated LD_50_ gives prior information regarding compound properties and would be used to select promising compounds and a starting dose for *in vivo* experiments. The evaluation of cytotoxicity of the abovementioned 3 types of amphiphilic compounds *in vitro* was assessed using the 3-(4,5-dimethylthiazol-2-yl)-2,5-diphenyltetrazolinium bromide (MTT) and crystal violet (CV) assays on two monolayer tumor cell lines, namely, HT-1080 (human fibrosarcoma) and MH-22A (mouse hepatoma). Additionally, the compound influence on “normal” mouse fibroblasts (NIH3T3) was estimated for the studies of structure-activity relationships and exploration of the effect of substituents. The results are presented in Tables 1–3. Studies of cytotoxicity of the amphiphilic self-assembling compounds revealed certain regularities.

### 3.4. Analysis of Structure-Activity Relationships

#### 3.4.1. Two Cationic Moieties Containing 1,4-DHP Amphiphiles (Table 1)

*(1) Modification of Ester Alkyl Groups*. Compounds possessing short alkyl chains in the ester groups at positions 3 and 5 of the dihydropyridine ring and organic heterocyclic cations in the methylene groups in positions 2 and 6 had very low cytotoxicity in cancer cell lines HT-1080 and MH-22A and high calculated LD_50_ values (comp. **1**–**9**). It means that the compounds were almost inert to the noncancerous cell line NIH3T3 and cancer cell lines HT-1080 and MH-22A. This refers to several groups of the mentioned type of compounds possessing short alkyls in 3,5-ester substituents (methyl, ethyl, and also the more elongated propoxyethyl groups): comp. **9**, **1**, **6**, **8**, and **7**. Additionally, 3,5-bis(diethyloxycarbonyl)-1,4-DHP amphiphiles comprising substituted pyridinio moieties in positions 2 and 6 (compounds **2**–**5**) have moderate cytotoxicity, around 3300 mg/kg. In most cases, for the 3,5-bis(diethyloxycarbonyl)-1,4-DHP compounds, it was not possible to calculate IC_50_ values for the cancer cell lines HT-1080 and MH-22A due to rather low cytotoxicities; calculated LD_50_ values of compounds **1**–**9** were >2000 mg/kg (for NIH3T3 cells).

On the contrary, analogues comprising dodecyl chains in the ester groups at positions 3 and 5 and pyridinium moieties in positions 2 and 6 of the dihydropyridine ring (see comp. **10** versus comp. **1**, comp. **12** versus comp. **2**, comp. **13** versus comp. **4**) showed significant cytotoxicity towards cancer cell lines HT-1080 and MH-22A and still very low cytotoxicity in noncancerous NIH3T3 cells. For example, LD_50_ values for compounds **10**, **12**, and **13** were 1482, 1431, and 1706 mg/kg, respectively, while cytotoxicity towards HT-1080 cells was 3–10 *μ*g/ml and towards MH-22A cells was 3–40 *μ*g/ml. In principle, the obtained data coincide with observations on the impact of dihydropyridine on cell growth, where it was concluded that a long alkyl chain containing 1,4-DHP amphiphiles show promising dual activity—proliferation inhibition on cancer cell lines and proliferation stimulating effect on normal cell lines [31]. It should be noted that the 3,5-bis(dodecyloxycarbonyl)-1,4-DHP amphiphile **14** with an electron-withdrawing acetyl group in the pyridinium moieties in positions 2 and 6 of the 1,4-DHP ring was practically nontoxic on noncancerous NIH3T3 cells and demonstrated selective cytotoxicity toward cancer cells and pronounced cytotoxicity of around 3 *μ*g/ml on HT-1080 cells and of 50-100 *μ*g/ml on MH-22A cells.

Substitution of fluorine for hydrogen atoms in alkyl chains of the 1,4-DHP amphiphiles decreased cytotoxicity of the abovementioned compounds. Thus, substitution of the 3,5-dodecyloxycarbonyl alkyl chain's most distant hydrogen atom by trifluoromethyl groups (comp. **22** versus comp. **10**) leads to lower cytotoxicity to cancer cell lines HT-1080 (10–18 *μ*g/ml versus 3 *μ*g/ml) and MH-22A (10–19 *μ*g/ml versus 3–6 *μ*g/ml) and to higher cytotoxicity to noncancerous cell line NIH3T3 (771 mg/kg versus 1482 mg/kg). In this case, the influence of the anion was not taken into account, but that also could give an effect. Substitution of seven methylene groups of a 3-dodecyloxy moiety by seven difluoromethylene groups leads to the formation of an unsymmetrical 1,4-DHP amphiphile **23**, which has lower cytotoxicity to the studied cancer and normal cell lines (comp. **23** versus comp. **10**)—a cytotoxicity of around 47–75 *μ*g/ml versus 3–6 *μ*g/ml on cancer cell lines and LD_50_ 3448 mg/kg versus 1482 mg/kg, respectively. Further substitution of the next seven methylene groups by seven difluoromethylene groups (comp. **24**) leads to subsequent diminishing of cytotoxicity to cancer cell lines (around 100 *μ*g/ml) and also low calculated toxicity to noncancerous cell line NIH3T3 (>2000 mg/kg). In the case of the more extended partially fluorinated 3,5-heptadecafluorononadecyl chains (comp. **25**), the compounds revealed undetectable toxicity to the two cancer cell lines and also low toxicity to the noncancerous cell line NIH3T3. So, from the obtained results (Table 1), it is evident that the perfluorinated 1,4-DHP amphiphiles (compounds **23**–**25**) are nontoxic in the tested cell lines.

*(2) Modification of Substituents at Position 4 of the 1,4-DHP Ring*. Variations of several types of substituents in position 4 of the DHP ring of 3,5-bis(diethyloxycarbonyl)-1,4-DHPs were performed. Compounds with phenyl (comp. **1**–**5**), substituted phenyl (2-difluoromethylphenyl (comp. **7** and **9**), trifluoromethyl (comp. **6**)), and ethoxycarbonyl (comp. **8**) substituents were obtained, but no significant influence on the cytotoxicity of the tested 1,4-DHP amphiphiles was observed. Introducing an HO-substituent in a phenyl moiety in position 4 of the 1,4-DHP ring gives comp. **18** and **19** which are 4-(4′-hydroxyphenyl) analogues of corresponding 4-phenyl-DHPs **10** and **12**, respectively. It was demonstrated that the introduction of an HO-substituent does not give a strong influence on the cytotoxicity of the compounds.

*(3) Modification of the Cationic Moieties*. Modification of the cationic moieties in positions 2 and 6 of the 1,4-DHP ring (in the case of 3,5-dodecyloxycarbonyl substituents) may result in substantially different toxicological properties. The insertion of substituents in the pyridinium ring leads to some quantitative modifications of cytotoxicity—mainly to slightly diminished cytotoxicity in the case of 4-methyl and 4-dimethylamino substituents (comp. **12** and comp. **13**)—while introducing a 3-acyl substituent (comp. **14** versus comp. **10**) in the pyridinium moiety did not give any influence on the cytotoxicity in HT-1080 cells (in both cases around 3 *μ*g/ml), but decreased cytotoxicity in MH-22A cells (50–100 *μ*g/ml and 3–6 *μ*g/ml, respectively) and also decreased cytotoxicity in noncancerous cell line NIH3T3 (4040 mg/kg versus 1482 mg/kg). Comparison of the cytotoxicity of the compounds with 4-methylpyridinium and 4-trifluoromethyl pyridinium moieties (comp. **12** versus comp. **21**) showed that there are no significant changes of cytotoxicity in cancer HT-1080 and MH-22A cells (5–10 *μ*g/ml versus 2–4 *μ*g/ml, and 29–40 *μ*g/ml versus 19–49 *μ*g/ml, respectively) but a twofold increase of cytotoxicity in noncancerous NIH3T3 cells (1431 mg/kg versus 619 mg/kg). Introduction of a pyrazinium moiety instead of a pyridinium moiety (comp. **16** versus comp. **10**) gave a compound which was practically inert to the studied cancer cell lines HT-1080 and MH-22A and was also nontoxic to normal NIH3T3 cells. Exchange of the heteroaromatic pyridinium moieties to saturated heterocyclic moieties—N-methylmorpholinium fragments (comp. **15**)—led to slightly lower anticancer activity of the compound towards the abovementioned cancer cell lines and comparatively higher cytotoxicity to NIH3T3 cells (979 mg/kg). Also, in this case, the influence of the anion was not taken into account, but it may give an effect (ClO_4_^−^ instead of Br^−^). Introducing N,N-dimethylcyclohexylammonium moieties as the cationic part of the amphiphile gave compound **17** with less cytotoxicity (23–54 *μ*g/ml) on both tested cancer cell lines and an LD_50_ value of 1274 mg/kg, while the LD_50_ value of compound **18** with the introduced aliphatic N,N-dimethyl-N-dodecylammonium moieties was 836 mg/kg. It was shown that compound **18** with aliphatic ammonium fragments was more cytotoxic. The obtained data is in agreement with conclusions by Lv et al. that among the synthetic cationic delivery systems, quaternary ammonium surfactants are more toxic than their analogues with the cationic charge delocalized in a heterocyclic system [45].

*(4) Change of the Anions*. Insertions of the BF_4_^−^ anion instead of the usual Br^−^ anion (comp. **11** versus comp. **10**) lead to an increase in basal toxicity—1053 and 1482 mg/kg, respectively—but a decrease in cytotoxicity on HT-1080 (around 30 *μ*g/ml and 3 *μ*g/ml, respectively) and MH-22A (30 *μ*g/ml and 3–6 *μ*g/ml, respectively) cell lines.

*(5) Change of Dehydrogenation Degree*. Lipid-like pyridine derivative **27**, as the oxidized form of compound **10**, demonstrated very close cytotoxicity data to the corresponding dihydro compound **10**, in all cases around 3 *μ*g/ml, while basal toxicity on noncancerous cell line NIH3T3 is significantly diminished: comp. **27** is not harmful at all (LD_50_ is 3948 mg/kg). In this case also, the influence of the anion was not taken into account, but it may give an effect (ClO_4_^−^ instead of Br^−^).

*(6) The Duplication of Moieties*. Synthetic lipid-like compound **26** was obtained as a “dimeric” form of compound **10**, which was proposed as a promising DNA delivery agent. Cytotoxicity data of amphiphiles (comp. **26** versus comp. **10**) demonstrated that this structural modification slightly decreased cytotoxicity of the target compound **26** in both tested cancer HT-1080 and MH-22A cells (12–27 *μ*g/ml versus 3–6 *μ*g/ml, respectively) and also considerably decreased the LD_50_ value—5164 mg/kg versus 1482 mg/kg.

#### 3.4.2. Cationic Moiety Containing 3,4-Dihydro-2(1*H*)-pyridones (Table 2)

*(1) Modification of Ester Alkyl Group*. Same as in the case of 1,4-DHP amphiphiles also, pyridones with a short alkyl chain—the methyl group in the ester moiety and pyridinium in the cationic part of the molecule (comp. **28** and **29**)—were inert to tested cancer cells HT-1080 and MH-22A and demonstrated high calculated LD_50_ values > 2000 mg/kg. In the case of comp. **29**, the influence of the N-substituent in the pyridone and, in the case of comp. **28**, the influence of substituent in the phenyl moiety at position 4 of the pyridone cycle were not taken into account. Two other methyl esters—comp. **33** and **36**—demonstrated significant cytotoxicity on all the tested cell lines. This could be explained by the influence of the cationic moiety in the compound.

Substitution of fluorine for hydrogen atoms in the alkyl chain of the pyridone amphiphiles did not give a strong influence on the cytotoxicity of the compounds, and it seems that this also was dependent on the cationic moiety and other substituents of the compound. So, in the case of 4-unsubstituted pyridones with the N,N-dimethylcyclohexylammonium moiety, comp. **37** with hydrogen atoms in the ester moiety and comp. **38** with fluorine atoms in the ester moiety demonstrated similar cytotoxicity in all of the tested cell lines, while for cytotoxicity of 4-phenyl pyridones with N,N-dimethylcyclohexylammonium moiety, comp. **34** with hydrogen atoms in the ester moiety versus comp. **35** with fluorine atoms in the NIH3T3 cells was two times higher (346 mg/kg versus 898 mg/kg, respectively).

*(2) Modification of Substituents at Position 4 of 3,4-Dihydro-2(1H)-pyridone Ring*. The series of 3,4-dihydro-2(1*H*)-pyridones with a phenyl substituent at position 4 of the pyridone ring and also the 4-unsubstituted ones were compared. In the case of 4-phenyl pyridone **34** and 4-unsubstituted pyridone **37**, both compounds containing the same N,N-dimethylcyclohexylammonium moiety and hydrogen atoms in the alkyl chain had no significant change of cytotoxicity on the tested cell lines. While in the case of 4-phenyl pyridone **35** and 4-unsubstituted pyridone **38**, both compounds containing the same N,N-dimethylcyclohexylammonium moiety and fluorine atoms in the alkyl chain, the 4-unsubstituted **38** possessed a slightly higher cytotoxicity—LD_50_ values were 553 mg/kg for comp. **38** and 898 mg/kg for comp. **35**.

*(3) Change of the Anion*. Change to perchlorate from the usual bromine anion (comp. **32** versus comp. **31**) did not give any influence on cytotoxicity on the tested cell lines.

*(4) Modification of the Cationic Moiety*. It is demonstrated that the dihydropyridone amphiphile **30** with a triphenylphosphonium polar head group was about 3 times less cytotoxic than the analogous dihydropyridone **31** with a pyridinium head group in the NIH3T3 cells, i.e., 1779 mg/kg versus 604 mg/kg. Change of pyridinium to N,N-dimethylcyclohexylammonium moiety as the cationic head group of dihydropyridone—comp. **31** versus comp. **35**—led to a slightly diminished cytotoxicity on cells in the case of comp. **35**. In this case, we observed the opposite relationship as for 1,4-DHP amphiphiles where it was demonstrated that quaternary ammonium surfactants are more toxic than their heterocyclic analogues. Toxicity of cationic lipids may be connected with the structure of their head groups [41].

The obtained data demonstrated that the 3,4-dihydro-2(1*H*)-pyridone amphiphiles (Table 2) with a pyridinium polar head group and introduced fluorine atoms in the ester moiety (comp. **31** and comp. **32**) were substantially more cytotoxic to tested cells than the structurally related 1,4-DHP amphiphiles **23** and **24**.

Most dihydropyridone series cationic amphiphiles show marked cytotoxicity towards cancer cells and medium cytotoxicity towards normal NIH3T3 cells: compounds possessing 1 or 2 long alkyl chains in ester and/or ammonium groups (with or without fluorine atoms on alkyl chains (comp. **31**–**34** and **36**–**38**)). There is an exclusion: comp. **30** possessing the triphenylphosphonium cationic group.

The obtained data allows one to choose compounds for putative use depending on their structure. Thus, due to the polyfluorinated alkyl ester groups in the DHP molecule, low toxicity (practically inert) amphiphilic compounds can be obtained (comp. **25**). Inert amphiphilic self-assembling compounds could be used as materials to form gene transfection or drug delivery nanoparticles for transmembrane transport according to the paradigm: pharmacologically inert transport vehicles should be used.

#### 3.4.3. 2-Amino-3-alkoxycarbonylalkylammonium Halides (Table 3)

The last group of delivery systems was formed by a 2-amino-3-alkoxycarbonylalkylammonium cationic moiety containing derivatives **39**–**48**.

*(1) Modification of the Cationic Moiety*. The cationic moiety was changed for 2-amino-3-hexadecyloxycarbonylammonium derivatives, namely, comp. **39**–**43**, and **45**. The obtained data demonstrated that N-(2-amino-4-(hexadecyloxy)-4-oxobut-2-en-1-yl)-N,N-dimethylcyclohexanaminium chloride (comp. **39**) shows the highest cytotoxicity in all the tested cell lines with a LD_50_ value of 97 mg/kg, while alkyl moiety containing compounds—N-(2-amino-4-(hexadecyloxy)-4-oxobut-2-en-1-yl)-N,N-dimethylhexan-1-aminium chloride (comp. **43**) and N-(2-amino-4-(hexadecyloxy)-4-oxobut-2-en-1-yl)-N,N-dimethyldodecan-1-aminium chloride (comp. **45**)—demonstrated lower cytotoxicity in all the tested cell lines with LD_50_ values of 538 and 573 mg/kg, respectively.

*(2) Modification of the Ester Alkyl Groups*. The influence of the alkyl moiety was compared for two amphiphiles—comp. **48** with decyl ester versus comp. **45** with hexadecyl ester. The obtained LD_50_ data show that the difference is not large—485 mg/kg versus 573 mg/kg, respectively.

*(3) Change of the Anion*. The anions from the usual bromide comp. **44** were changed to chloride and also to iodide (comp. **45** and comp. **46**). According to IC_50_ and LD_50_ data, the change of the anion did not have any influence on the cytotoxicity in NIH3T3 cell lines; IC_50_ values were around 30 *μ*g/ml.

### 3.5. Electrochemical Oxidation

The electrochemical oxidation of various 1,4-dihydropyridine derivatives has been extensively studied [46–50] including 1,4-DHP derivatives containing cationic moieties [29, 44]. The electrochemical oxidation of the selected compounds studied in this work was performed by cyclic voltammetry on a stationary glassy carbon electrode in dry acetonitrile; the data is presented in Table 4. The perchlorates of the tested amphiphiles were obtained from the corresponding bromides by treating their abs. MeOH solutions with excess of conc. HClO_4_ according to the procedure elaborated by Turovska et al. [44].

Now, we have used electrooxidation potentials to characterize electron donor properties of the studied compounds.

Compounds from the 1st group, containing cationic pyridinium methylene groups in positions 2 and 6 in the 1,4-DHP ring, have electrooxidation potentials of 1.57–1.58 V both in the case of 3,5-diethoxycarbonyl- and 3,5-didodecyloxycarbonyl-1,4-DHPs (comp. **1** and comp. **10**). This is also in agreement with our previous results, where the electrochemical oxidation potential of comp. **1** was determined as 1.7 V and electrochemical oxidation of this compound was demonstrated as a two-electron process [29]. Introduction of the CF_3_ groups at the *ɷ*-carbon atom of a dodecyl chain in the ester moieties (comp. **18**) does not change the value of the electrooxidation potential, which is also 1.57 V. On the contrary, the addition of a CF_3_ group in the pyridinium moiety in the 2 and 6 positions of the 1,4-DHP cycle (comp. **21**) leads to a slight increase of the electrooxidation potential (1.63 V). Moreover, a change of the 17 terminal H atoms to F atoms in one or both dodecyl chains leads to a further increase of the electrooxidation potential of 1.69–1.70 V (comp. **23** and comp. **24**). It should be noted that the parent compounds—1,4-DHP derivatives without cationic moieties—demonstrated lower electrooxidation potentials. Thus, 4-phenyl-substituted Hantzsch 1,4-dihydropyridine has a 1.08 V potential on a glassy carbon electrode [50] and the other 4-aryl-substituted 1,4-DHPs have 1.11 V potentials [51], but 4-monoalkyl-substituted 1,4-dihydropyridines at the same conditions have 1.01–1.03 V oxidation potentials [47].

Compounds from the 2nd group—amphiphilic 3,4-dihydropyridone derivatives with a pyridinium methylene moiety in position 6—have more positive electrooxidation potential. So, the unsubstituted at position 4 pyridone derivative **29** has an electrooxidation potential of 2.35 V. A compound possessing a phenyl substituent at position 4 and a 5-heptadecylfluorododecylcarboxy moiety in position 5 (comp. **38**) has a slightly lower oxidation potential (2.04 V), while the parent 4-unsubstituted or 4-phenyl-substituted 3,4-dihydropyridone derivatives without cationic moiety in position 6 have electrooxidation potentials of 1.52–1.64 V [52].

Compounds from the 3rd group—tested open-chain 2-amino-3-alkoxycarbonylalkylammonium halides **42** and **44**—have oxidation potentials of 1.49 V and 1.24 V, respectively.

Compounds from the 1st group (comp. **1**–**24**, Table 1) could be considered as analogues of 1,4-dihydronicotinamide and model compounds of redox coenzyme NAD(P)H. Many 4-aryl-1,4-DHPs possess antioxidant properties, including several Ca^2+^ channel blockers [53]. The antiradical activity (ARA) of two 1,4-DHPs containing cationic moieties was determined by a 1,1-diphenyl-2-picrylhydrazyl (DPPH) radical assay; the results were expressed as a percentage (%) of the DPPH free radical scavenging, and the untreated level of the DPPH radical was designated as 100% [13, 31]. It was demonstrated that 3,5-didodecyloxycarbonyl-4-phenyl-1,4-dihydropyridine derivatives containing pyridinium moieties showed 25–60% radical scavenging activity which are comparable with the ARA of Diludin [54] (40%)—a widely known antioxidant. Other 1,4-DHP amphiphiles containing saturated heterocyclic moieties—N-methylmorpholinium or N-methylpyrrolidinium derivatives—demonstrated more pronounced ARA, 95% and 54%, respectively. For 1,4-DHP amphiphiles possessing pyridinium moieties, the positive charge is delocalized in the heteroaromatic cycle, causing ARA reduction; for example, the electron donor dimethylamino group as a substituent of pyridinium moiety leads to a lower ARA (27%, comp. **13**) [13].

### 3.6. Determination of Log*P* and Molecular Topological Polar Surface Area

The lipophilicity of molecules represents their affinity for a lipophilic environment, and the lipophilicity may be expressed as log*P* [55, 56]. The molecular polar surface area (PSA) is a very useful parameter for the prediction of drug transport properties, and PSA is defined as a sum of the surfaces of polar atoms [57]. In practice, medicinal chemists use the PSA to quantify the polarity of drug molecules [58]. Data of the calculated topological polar surface area represent the compound's blood-brain barrier permeability [59, 60]. It allows one to plan further activities for the pleiotropic compounds.

Lipid-type compounds could be used as biologically active compounds *per se* or as transport vehicles or additives, so their lipophilicity (log*P*) and topological polar surface area (TPSA) were calculated, and the data are recorded in Table 5.

Log*P* values which surpass 5, according to Lipinski's Rule of Five, characterize compounds as lipophilic [61]. According to the obtained data for lipid-like amphiphiles, in some cases, the values of log*P* were <5, in particular for compounds **1**, **2**, **4**, **6**, **7**, **28**, and **29**. These compounds comprised short alkyl moieties in the ester groups. Log*P* values close to 5 were obtained for compounds **36**, **41**, **42**, and **47**, namely, 4.70, 5.63, 5.44, and 5.40, respectively. Log*P* values for dicationic 1,4-DHP amphiphiles possessing longer alkyl chains or fluorinated alkyl groups in the ester moieties were determined in the 11–24.5 interval, while for long alkyl ester moieties containing 3,4-dihydropyridone amphiphiles, log*P* values were determined in the 6–15 interval. The difference could be due to the number of alkyl groups. Log*P* values for other open-chain com12pounds were determined in the 6–11 interval.

Nevertheless, TPSA never surpasses 90, so the compounds are prone to permeate cells; additionally, they can penetrate the blood-brain barrier [62]. Among all tested amphiphiles, only for 1,1′-((4-(2-(difluoromethoxy)phenyl)-3,5-bis((propoxymethoxy)carbonyl)-1,4-dihydropyridine-2,6-diyl)bis(methylene))bis(pyridin-1-ium) dibromide (comp. **7**) was the TPSA value higher than 90, i.e., 98.34 Å^2^. This could be explained by the influence of the structure components of the compound. TPSA values for the other compounds were defined in accordance with the main fragments of the compound structures. So, for the other 1st group compounds—dicationic 1,4-DHP amphiphiles—TPSA values were in the 71–77 Å^2^ interval; for the 2nd group compounds—cationic 3,4-dihydropyridone amphiphiles—TPSA values were in the 50–58 Å^2^ interval with the exception of compound **28** which had a TPSA value of 67.64 Å^2^; and for the 3rd group compounds—open-chain 2-amino-3-alkoxycarbonylalkylammonium cationic moiety containing amphiphiles—the TPSA values were in the 52–56 Å^2^ interval.

## 4. Conclusions

Polyfunctional self-assembling synthetic lipid-like compounds, such as pharmacological and chemotherapeutical agents, namely, 3,5-dialkoxycarbonyl-1,4-dihydropyridines (1,4-DHPs) comprising pyridinium or ammonium substituents at the 2 and 6 positions; structurally related compounds, derivatives of 3,4-dihydro-2-oxopyridines as isomeric 3,4-dihydropyridine structures with an additional intracyclic carbonyl group; and the N-*β*-carbonylvinylamido system, namely, 2-amino-3-alkoxycarbonylalkylammonium halides as open chain analogues of the first type of the abovementioned compounds, were studied. The main properties and major functions of these compounds are their amphiphilic character, liposome-forming ability, RNA transfection (by self-assembling compounds), antiradical and antioxidant properties, growth regulation—both in malignant and nonmalignant cell types—anticancer properties due to cytotoxicity, and MDR inhibition [13, 16, 27, 28, 31, 33].

In this work we have demonstrated biological properties of cationic 1,4-dihydropyridine as self-assembling synthetic lipids and dihydropyridones as well as open-chain analogues: their cytotoxicity against cancer cell lines HT-1080 and MH-22A in comparison with cytotoxicity against normal NIH3T3 cells. The obtained data showed that 1,4-DHP derivatives containing cationic moieties in positions 2 and 6 and possessing dodecyl alkyl chains in the ester groups in positions 3 and 5 demonstrated high cytotoxicity on cancer cells HT-1080 and MH-22A, but low cytotoxicity on noncancerous NIH3T3 cells. According to our previous studies, these compounds also demonstrated significant antiradical activity and also gene delivery activity [13], and for some of them, reversal of multidrug resistance in murine lymphoma cells [16]. Together with antiradical activity, cell growth regulation, multidrug resistance inhibition, nucleic acid delivery, and the polyfunctional (pleiotropic) type of properties of the mentioned compounds open new avenues for their studies and use. According to literature data, liposomes could be used not only to transport biologically active compounds but also to have their own specific biological activity, e.g., to protect cells and encapsulated components against oxidative damage. Liposomes are proposed for the delivery of antioxidants for protection against pathological conditions related to oxidative stress [63]. In our case, liposomes could be used *per se* to protect against oxidative damage.

A calculated degree of lipophilicity and TPSA data can be used to choose compounds according to their permeability through membranes, including the blood-brain barrier, to guide them to the proper location. It was demonstrated that membrane permeability in a variety of systems, including model liposome bilayers, various cells, and epidermal tissue, correlated strongly with data regarding hydrocarbon-water partition coefficients [64]. TPSA values for selected compounds were defined in accordance with the main fragments of compound structures. The determined log*P* values were highest for dodecyl ester groups in positions 3 and 5 of the 1,4-DHP and lowest for short alkyl chain containing amphiphiles.

This study also revealed the correlation of the cytotoxic effects of 3 groups of structurally related synthetic cationic lipids according to their molecular structures. The results indicated that among the tested compound groups, amphiphiles based on the 1,4-DHP core demonstrated high cytotoxicity in cancer cells HT-1080 and MH-22A, but low cytotoxicity in the noncancerous NIH3T3 cells.

The obtained results may serve as guidelines for the development of drug formulations to be used in cancer treatment on the basis of these pleiotropic lipid-like 1,4-DHP amphiphiles.

## Figures and Tables

**Table 1:** Structure, cytotoxicity, and calculated basal toxicity of 1,4-dihydropyridines containing cationic moieties. 
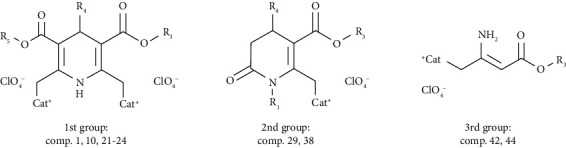

Comp.	Cat^+^	R^3^	R^4^	R^5^	X^−^	HT-1080		MH-22A		NIH3T3		Ref.
						IC_50_ (CV) (*μ*g/ml)	IC_50_ (MTT) (*μ*g/ml)	IC_50_ (CV) (*μ*g/ml)	IC_50_ (MTT) (*μ*g/ml)	IC_50_ (NR) (*μ*g/ml)	LD_50_ (mg/kg)	
1	Py	C_2_H_5_	Ph	C_2_H_5_	Br	∗	∗	∗	∗	∗	>2000	[29]
2	Py-Me-4	C_2_H_5_	Ph	C_2_H_5_	Br	∗	>740	∗	∗	∗	>3360	[30]
3	Py-NH_2_-4	C_2_H_5_	Ph	C_2_H_5_	Br	nt	441 ± 46	nt	>740	∗	>3360	Suppl.
4	Py-NMe_2_-4	C_2_H_5_	Ph	C_2_H_5_	Br	nt	324 ± 29	nt	608 ± 49	∗	>3500	Suppl.
5	Py-Me-3	C_2_H_5_	Ph	C_2_H_5_	Br	nt	>740	nt	∗	∗	>3360	Suppl.
6	Py	C_2_H_5_	C_6_H_4_-CF_3_-2	C_2_H_5_	Br	∗	∗	∗	∗	∗	>2000	[20]
7	Py	C_2_H_4_OC_3_H_7_	C_6_H_4_-OCHF_2_-2	C_2_H_4_OC_3_H_7_	Br	∗	∗	∗	∗	∗	>2000	[20]
8	Py	C_2_H_5_	COOCH_3_	C_2_H_5_	I	∗	∗	∗	∗	∗	>2000	[20]
9	N-Me-morph	CH_3_	C_6_H_4_-OCHF_2_-2	CH_3_	ClO_4_	∗	∗	∗	∗	∗	>2000	[31]
10	Py	C_12_H_25_	Ph	C_12_H_25_	Br	3 ± 0.5	3 ± 0.3	6 ± 1	3 ± 0.8	100 ± 6	1482	[13, 28]
11	Py	C_12_H_25_	Ph	C_12_H_25_	BF_4_	31 ± 4	28 ± 9	30 ± 6	30 ± 9	47 ± 8	1053	Suppl
12	Py-Me-4	C_12_H_25_	Ph	C_12_H_25_	Br	10 ± 2	5 ± 1	40 ± 2	29 ± 2	79 ± 11	1431	[13]
13	Py-NMe_2_-4	C_12_H_25_	Ph	C_12_H_25_	Br	10 ± 1	3 ± 0.6	6 ± 2	10 ± 3	119 ± 13	1706	[13]
14	Py-C(=O)CH_3_-3	C_12_H_25_	Ph	C_12_H_25_	Br	3 ± 0.4	3 ± 0.3	100 ± 13	49 ± 9	922 ± 24	4040	[13]
15	N-Me-morph	C_12_H_25_	Ph	C_12_H_25_	ClO_4_	13 ± 3	12 ± 3	34 ± 11	27 ± 9	35 ± 13	979	[13]
16	Pyr	C_12_H_25_	Ph	C_12_H_25_	Br	∗	∗	∗	∗	∗	>2000	[13]
17	NMe_2_CyHex	C_12_H_25_	Ph	C_12_H_25_	Br	35 ± 9	23 ± 3	54 ± 16	35 ± 9	59 ± 12	1274	Suppl
18	Py	C_12_H_25_	C_6_H_4_-OH-4	C_12_H_25_	Br	4.3 ± 0.6	10 ± 2	3.4 ± 0.6	16 ± 2	95 ± 7	1479	Suppl
19	Py-CH_3_-4	C_12_H_25_	C_6_H_4_-OH-4	C_12_H_25_	Br	32 ± 8	22 ± 5	21 ± 3	30 ± 8	44 ± 11	1087	Suppl
20	N-Me_2_C_12_H_25_	C_12_H_25_	Ph	C_12_H_25_	Br	3 ± 0.3	3 ± 0.4	10 ± 2	10 ± 2	19 ± 6	836	Suppl
21	Py-CF_3_-4	C_12_H_25_	Ph	C_12_H_25_	Br	2 ± 0.3	4 ± 0.5	49 ± 8	19 ± 6	12 ± 4	619	[32]
22	Py	C_12_H_24_CF_3_	Ph	C_12_H_24_CF_3_	ClO_4_	18 ± 5	10 ± 2	10 ± 1	19 ± 5	16 ± 3	771	[32]
23	Py	C_12_H_25_	Ph	(CH_2_)_4_(CF_2_)_7_CF_3_	Br	50 ± 6	47 ± 8	47 ± 2	75 ± 11	477 ± 25	3448	Suppl
24	Py	(CH_2_)_4_(CF_2_)_7_CF_3_	Ph	(CH_2_)_4_(CF_2_)_7_CF_3_	Br	100 ± 11	100 ± 9	∗	∗	∗	>2000	Suppl
25	Py	(CH_2_)_11_(CF_2_)_7_CF_3_	Ph	(CH_2_)_11_(CF_2_)_7_CF_3_	Br	∗	∗	∗	∗	∗	>2000	Suppl
26	“Dimeric” form of comp. **10**				Br	12 ± 4	14 ± 2	27 ± 6	21 ± 4	717 ± 56	5164	Suppl
27	Oxidated form of comp. **10**				ClO_4_	3.2 ± 0.8	3.1 ± 0.5	3.0 ± 0.3	3.3 ± 0.4	900 ± 42	3946	[14]

nt: not tested; ∗: not detected.

**Table 2:** Structure, cytotoxicity, and calculated basal toxicity of 3,4-dihydro-2(1*H*)-pyridones containing cationic moiety. 
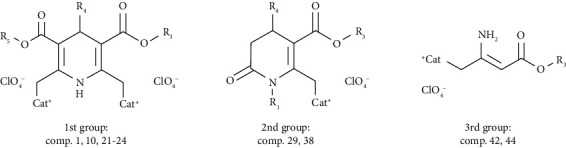

Comp.	R^1^	Cat^+^	R^3^	R^4^	X^−^	HT-1080		MH-22A		NIH3T3		Ref.
						IC_50_ (CV) (*μ*g/ml)	IC_50_ (MTT) (*μ*g/ml)	IC_50_ (CV) (*μ*g/ml)	IC_50_ (MTT) (*μ*g/ml)	IC_50_ (NR) (*μ*g/ml)	LD_50_ (mg/kg)	
28	H	Py	CH_3_	C_6_H_4_-OCHF_2_-2	Br	∗	∗	>100	∗	1132 ± 85	>2000	Suppl
29	PhCH_2_	Py	CH_3_	H	Br	∗	∗	∗	∗	972 ± 27	>2000	Suppl
30	H	PPh_3_	(CH_2_)_4_(CF_2_)_7_CF_3_	Ph	Br	10 ± 2	9 ± 2	30 ± 11	39 ± 5	127 ± 11	1779	[33]
31	H	Py	(CH_2_)_4_(CF_2_)_7_CF_3_	Ph	Br	3 ± 0.3	3 ± 0.2	3 ± 1	3 ± 0.6	15 ± 2	604	[33]
32	H	Py	(CH_2_)_4_(CF_2_)_7_CF_3_	Ph	ClO_4_	3 ± 0.3	3 ± 0.5	2 ± 0.3	3 ± 0.2	15 ± 3	618	Suppl
33	H	N-Me_2_C_12_H_25_	CH_3_	Ph	Br	2 ± 0.4	1 ± 0.2	2 ± 0.1	2 ± 0.6	4 ± 1	269	Suppl
34	H	N-Me_2_C_12_H_25_	C_12_H_25_	Ph	Br	2 ± 0.2	2 ± 0.6	3 ± 0.8	<1	6 ± 2	346	Suppl
35	H	N-Me_2_C_12_H_25_	(CH_2_)_4_(CF_2_)_7_CF_3_	Ph	Br	3 ± 0.3	30 ± 11	26 ± 6	30 ± 8	27 ± 9	898	Suppl
36	H	N-Me_2_C_12_H_25_	CH_3_	H	Br	2 ± 0.2	1 ± 0.2	18 ± 3	16 ± 3	63 ± 11	831	Suppl
37	H	N-Me_2_C_12_H_25_	C_12_H_25_	H	Br	2 ± 0.5	3 ± 0.4	3 ± 0.6	1 ± 0.2	7 ± 1	369	Suppl
38	H	N-Me_2_C_12_H_25_	(CH_2_)_4_(CF_2_)_7_CF_3_	H	Br	3 ± 0.2	2 ± 0.2	4 ± 0.8	<1	11 ± 3	553	Suppl

∗: not detected.

**Table 3:** Structures, cytotoxicity, and calculated toxicity of 2-amino-3-alkoxycarbonylalkylammonium halides. 
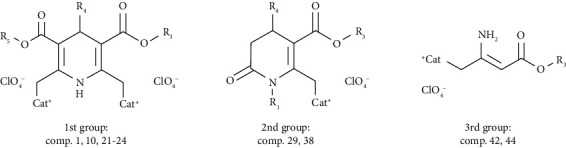

Comp.	Cat^+^	R	X^−^	HT-1080		MH-22A		NIH3T3		Ref.
				IC_50_ (CV) (*μ*g/ml)	IC_50_ (MTT) (*μ*g/ml)	IC_50_ (CV) (*μ*g/ml)	IC_50_ (MTT) (*μ*g/ml)	IC_50_ (NR) (*μ*g/ml)	LD_50_ (mg/kg)	
39	N-Me_2_CyHex	C_16_H_33_	Cl	0.35 ± 0.06	0.5 ± 0.1	1 ± 0.6	0.2 ± 0.06	0.7 ± 0.1	97	Suppl
40	N-Me(CH_2_)_4_	C_16_H_33_	Cl	<1	<1	1 ± 0.2	<1	8 ± 1	312	Suppl
41	N-Me_2_(CH_2_)_2_NMe_2_	C_16_H_33_	Cl	2 ± 0.3	2 ± 0.6	1 ± 0.3	1 ± 0.2	5 ± 1	286	Suppl
42	N-Me(CH_2_)_2_N(CH_2_)_2_Me	C_16_H_33_	Cl	2 ± 0.4	2 ± 0.2	1 ± 0.1	2 ± 0.3	4 ± 1	237	Suppl
43	N-Me_2_C_6_H_13_	C_16_H_33_	Cl	3 ± 0.6	2 ± 0.4	2 ± 0.2	1 ± 0.2	21 ± 6	538	Suppl
44	N-Me_2_C_12_H_25_	C_16_H_33_	Br	3 ± 0.4	3 ± 0.3	25 ± 3	28 ± 6	31 ± 8	697	Suppl
45	N-Me_2_C_12_H_25_	C_16_H_33_	Cl	nt	nt	nt	nt	25 ± 9	573	Suppl
46	N-Me_2_C_12_H_25_	C_16_H_33_	I	nt	nt	nt	nt	34 ± 8	637	Suppl
47	Py	C_12_H_25_	I	3 ± 0.4	nt	2 ± 0.1	nt	14 ± 9	403	Suppl
48	N-Me_2_C_12_H_25_	C_10_H_21_	Cl	3 ± 0.4	nt	2 ± 0.2	nt	17 ± 3	485	Suppl

nt: not tested.

**Table 4:** Oxidation potentials (E^ox^) of selected compounds.
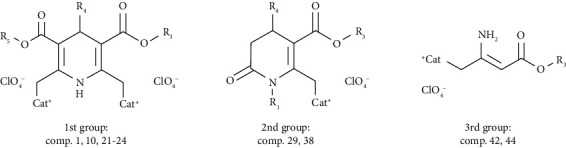

Group	Comp.	R^1^	R^3^	R^4^	R^5^	Cat^+^	E^ox^ (V)
1st	**1** ^∗^	—	C_2_H_5_	Ph	C_2_H_5_	Py	1.57
	**10** ^∗^	—	C_12_H_25_	Ph	C_12_H_25_	Py	1.58
	**21** ^∗^	—	C_12_H_25_	Ph	C_12_H_25_	Py-CF_3_-4	1.63
	**22**	—	C_12_H_24_CF_3_	Ph	C_12_H_24_CF_3_	Py	1.57
	**23** ^∗^	—	C_12_H_25_	Ph	(CH_2_)_4_(CF_2_)_7_CF_3_	Py	1.70
	**24** ^∗^	—	(CH_2_)_4_(CF_2_)_7_CF_3_	Ph	(CH_2_)_4_(CF_2_)_7_CF_3_	Py	1.69
2nd	**29** ^∗^	PhCH_2_	CH_3_	H	—	Py	2.35
	**38** ^∗^	H	(CH_2_)_4_(CF_2_)_7_CF_3_	Ph	—	Py	2.04
3rd	**42** ^∗^	—	C_16_H_33_	—	—	N-Me(CH_2_)_2_N(CH_2_)_2_Me	1.49
	**44** ^**#**^	—	C_16_H_33_	—	—	N-Me_2_C_12_H_25_	1.24

^∗^Original compounds—bromide; ^#^original compound—chloride.

**Table 5:** Log*P* and molecular topological polar surface area (TPSA) of selected compounds.

1st group			2nd group			3rd group		
Comp.	Log*P*	TPSA (Å^2^)	Comp.	Log*P*	TPSA (Å^2^)	Comp.	Log*P*	TPSA (Å^2^)
1	2.26	70.65	28	1.44	67.64	39	7.52	52.32
2	3.13	70.65	29	1.18	49.62	40	6.25	52.32
4	2.71	77.13	30	14.32	55.40	41	5.63	55.56
6	3.18	70.65	31	9.43	58.41	42	5.44	55.56
7	3.20	98.34	32	9.43	58.41	43	7.98	52.32
10	10.97	70.65	33	6.19	55.40	44	10.65	52.32
12	11.63	70.65	34	10.01	55.40	47	5.40	55.33
13	11.42	77.13	35	14.81	55.40	48	7.98	52.32
17	13.55	64.63	36	4.70	55.40			
20	19.81	64.63	37	8.52	55.40			
21	12.81	70.65	38	13.32	55.40			
22	13.22	70.65						
23	15.77	70.65						
24	18.64	70.65						
25	24.68	70.65						

## Data Availability

The experimental data used to support the findings of this study are available from the corresponding authors upon request.

## Supplementary Materials

Supplementary Materials Scheme S1: synthesis of 1,4-dihydropyridine (1,4-DHP) amphiphiles **1**–**7**, **9**–**22**, and **26**. Scheme S2: synthesis of unsymmetrical 1,4-DHP amphiphile **23**. Scheme S3: synthesis of 3,4-dihydro-2(1*H*)-pyridone (DHPDO) amphiphiles **28**–**38**. Scheme S4: synthesis of 2-amino-3-alkoxycarbonylalkylammonium halides **39**–**48**. Physicochemical characterization of new compounds is provided.
