# Supplementary material for: Pleiotropic Properties of Amphiphilic Dihydropyridines, Dihydropyridones, and Aminovinylcarbonyl Compounds
Source: Oxid Med Cell Longev. 2020 Dec 31;2020:8413713. doi: 10.1155/2020/8413713 (PMC7790557; doi:10.1155/2020/8413713)
Supplement: Supplementary Materials — Scheme S1: synthesis of 1,4-dihydropyridine (1,4-DHP) amphiphiles 1–7, 9–22, and 26. Scheme S2: synthesis of unsymmetrical 1,4-DHP amphiphile 23. Scheme S3: synthesis of 3,4-dihydro-2(1H)-pyridone (DHPDO) amphiphiles 28–38. Scheme S4: synthesis of 2-amino-3-alkoxycarbonylalkylammonium halides 39–48. Physicochemical characterization of new compounds is provided. [file 8413713.f1.docx]

**Supplementary information file**

**Pleiotropic properties of amphiphilic dihydropyridines, dihydropyridones and aminovinylcarbonyl compounds**

Martins Rucins, Rufus Smits, Anda Sipola, Brigita Vigante, Ilona Domracheva, Baiba Turovska, Ruslan Muhamadejev, Karlis Pajuste, Mara Plotniece, Arkadij Sobolev, Gunars Duburs, and Aiva Plotniece

Correspondence should be addressed to Gunars Duburs; gduburs@osi.lv and Aiva Plotniece; aiva@osi.lv

**Compounds from Table 1.**

**Synthesis of 1,4-DHP amphiphiles 1–7, 9–22**

Synthesis of 2,6-bis(pyridinium) and 2,6-bis(ammonium) 1,4-DHP dihalides was performed according to Scheme S1.

**Scheme S1**. Synthesis of 1,4-dihydropyridine (1,4-DHP) amphiphiles **1–7**, **9–22, 26**.

Briefly, 3,5-bis(alkoxycarbonyl)-2,6-dimethyl-4-aryl-1,4-dihydropyridines (**C**) were obtained from the corresponding acetoacetic ester **B** (2.0 eq), aldehyde **A** (1.0 eq) and a source of ammonia (1.2 eq) in classical Hantzsch synthesis[[1**–**3](#_ENREF_1)]. Bromination of 2,6-methyl groups of 1,4-DHPs **C** was performed by N-bromosuccinimide (NBS) (2.0 eq) in methanol giving 2,6-di(bromomethyl)-3,5-bis(alkoxycarbonyl)-4-aryl-1,4-dihydropyridines **D** according to the procedure described by Rucins *et al.* [[4](#_ENREF_4)]. Treatment of 2,6-di(bromomethyl)-3,5-bis(alkoxycarbonyl)-4-aryl-1,4-dihydropyridines with corresponding N-containing heterocyclic compounds (2.2 eq) offered the target 1,4-DHP amphiphiles **1–7**, **9–22**.

^1^H-NMR spectra data and other physico-chemical parameters of the previously reported compounds were in agreement with those reported in the literature.

**1,1'-((3,5-Bis(ethoxycarbonyl)-4-phenyl-1,4-dihydropyridine-2,6-diyl)bis(methylene))-bis(pyridin-1-ium) dibromide (1)** was synthesized according to the literature [[5](#_ENREF_5)]:

**1,1'-((3,5-Bis(ethoxycarbonyl)-4-phenyl-1,4-dihydropyridine-2,6-diyl)bis(methylene))-bis(4-methylpyridin-1-ium) dibromide (2)** was synthesized according to the literature [[6](#_ENREF_6)]:

**General procedure for synthesis of compounds 3–5**

According to Scheme S1. Briefly, the corresponding 3,5-bis(ethoxycarbonyl)-2,6-dimethyl-4-phenyl-1,4-dihydropyridines (**1C**) were obtained from ethyl acetoacetate (**1B**) (2.0 eq), benzaldehyde **1A** (1.0 eq) and ammonium acetate (1.2 eq) by classical Hantzsch synthesis. Bromination of 2,6-methyl groups of 1,4-DHPs **1C** were performed by N-bromosuccinimide (NBS) (2.0 eq) in methanol giving 2,6-di(bromomethyl)-3,5-bis(ethoxycarbonyl)-4-phenyl-1,4-dihydropyridine **1D** according to procedure described by Plotniece *et al.* [[5](#_ENREF_5)] Treatment of 2,6-di(bromomethyl)-3,5-bis(alkoxycarbonyl)-4-aryl-1,4-dihydropyridines with corresponding pyridinium derivatives (2.2 eq) gave the target 1,4-DHP amphiphiles **3–5**.

**1,1'-((3,5-Bis(ethoxycarbonyl)-4-phenyl-1,4-dihydropyridine-2,6-diyl)bis(methylene))-bis(4-aminopyridin-1-ium) dibromide (3)**

Yield: 72%; Mp. 230-232°C decomp. ^1^H-NMR (DMSO-d_6_, 300 MHz, δ): 10.17 (s, 1H), 8.20 (br.s, 4H), 8.13 (d, 4H, *J* = 7.4 Hz), 7.31–7.12 (m, 5H), 6.81 (d, 4H, *J* = 7.4 Hz), 5.53 and 5.20 (AB-system, 4H, *J* = 14.4 Hz), 4.96 (s, 1H), 4.06 (q, *J* = 7.1 Hz, 4H), 1.12 (t, *J* = 7.1 Hz, 6H). Anal. calcd for C_29_H_33_N_5_O_4_Br_2_×H_2_O: C, 50.23; H, 5.09; N, 10.10; found: C, 49.87; H, 4.72; N, 9.78.

**1,1'-((3,5-Bis(ethoxycarbonyl)-4-phenyl-1,4-dihydropyridine-2,6-diyl)bis(methylene))-bis(4-(dimethylamino)pyridin-1-ium) dibromide (4)**

Yield: 69%; Mp. 234-236°C. ^1^H-NMR (CDCl_3_, 300 MHz, δ): 10.61 (s, 1H), 8.38 (d, 4H, *J* = 7.9 Hz), 7.33–7.15 (m, 5H), 7.01 (d, 4H, *J* = 7.9 Hz), 5.81 and 5.48 (AB-system, 4H, *J* = 13.7 Hz), 5.04 (s, 1H), 4.18–4.03 (m, 4H), 3.33 (s, 12H), 1.24 (t, J = 7.1 Hz, 6H). Anal. calcd for C_33_H_41_N_5_O_4_Br_2_×H_2_O: C, 52.88; H, 5.78; N, 9.34; found: C, 52.86; H, 5.63; N, 9.23.

**1,1'-((3,5-Bis(ethoxycarbonyl)-4-phenyl-1,4-dihydropyridine-2,6-diyl)bis(methylene))-bis(3-methylpyridin-1-ium) dibromide (5)**

Yield: 75%; Mp. 197-199°C. ^1^H-NMR (DMSO-d_6_, 300 MHz, δ): 10.29 (s, 1H), 8.87 (s, 2H), 8.81 (d, 2H, *J* = 6.1 Hz), 8.45 (d, 2H, *J* = 8.0 Hz), 8.01 (dd, 2H, *J* = 8.0, 6.1 Hz), 7.37–7.16 (m, 5H), 6.09 and 5.59 (AB-system, 4H, *J* = 14.7 Hz), 5.02 (s, 1H), 4.13 – 3.98 (m, 4H), 2.45 (s, 6H), 1.10 (t, *J* = 7.1 Hz, 6H) ppm. Anal. calcd for C_31_H_35_N_3_O_4_Br_2_×H_2_O: C, 53.85; H, 5.39; N, 6.08; found: C, 53.61; H, 5.08; N, 5.95.

**1,1'-((3,5-Bis(ethoxycarbonyl)-4-(2-(trifluoromethyl)phenyl)-1,4-dihydropyridine-2,6-diyl)bis(methylene))bis(pyridin-1-ium) dibromide (6)** was synthesized according to the literature [[7](#_ENREF_7)]:

**1,1'-((4-(2-(Difluoromethoxy)phenyl)-3,5-bis((propoxymethoxy)carbonyl)-1,4-dihydro­pyridine-2,6-diyl)bis(methylene))bis(pyridin-1-ium) dibromide (7)** was synthesized according to the literature [[7](#_ENREF_7)]:

**1,1'-((3,5-Bis(ethoxycarbonyl)-4-(methoxycarbonyl)-1,4-dihydropyridine-2,6-diyl)-bis(methylene))bis(pyridin-1-ium) dibromide (8)** was synthesized according to the literature [[7](#_ENREF_7)]:

**1,1'-((4-(2-(Difluoromethoxy)phenyl)-3,5-bis(methoxycarbonyl)-1,4-dihydropyridine-2,6-diyl)bis(methylene))bis(1-methylpiperidin-1-ium) diperchlorate (9)** was synthesized according to the literature [[8](#_ENREF_8)]:

**1,1'-((3,5-Bis((dodecyloxy)carbonyl)-4-phenyl-1,4-dihydropyridine-2,6-diyl)bis(met­hylene))bis(pyridin-1-ium) dibromide (10)** was synthesized according to the literature [[1](#_ENREF_1), [3](#_ENREF_3)]:

**1,1'-((3,5-Bis((dodecyloxy)carbonyl)-4-phenyl-1,4-dihydropyridine-2,6-diyl)bis(met­hylene))bis(pyridin-1-ium) ditetrafluoroborate (11)**

According to Scheme S1. 1,1'-((3,5-Bis((dodecyloxy)carbonyl)-4-phenyl-1,4-dihydropyridine-2,6-diyl)bis(methylene))bis(pyridin-1-ium) dibromide (**10**) (1.0 eq) was dissolved in dry acetonitrile after which NH_4_BF_4_ (2.1 eq) was added and reaction mixture stirred for 24h. Inorganic precipitate was filtered off, solvent was evaporated and the residue was recrystallized from ethanol to give comp. **11**.

Yield: 69%; Mp. 160°C decomp. ^1^H-NMR (DMSO-d_6_, 200 MHz, δ): 9.93 (br.s, 1H), 8.88 (d, 4H, *J* = 6.4 Hz), 8.56 (t, 2H, *J* = 6.4 Hz), 8.07 (t, 4H, *J* = 6.4 Hz), 7.33**–**7.20 (m, 5H), 6.06 and 5.47 (AB-system, 4H, *J* = 13.5 Hz), 5.00 (s, 1H), 3.98 (t, 4H, *J* = 6.6 Hz), 1.55**–**1.44 (m, 4H), 1.24**–**1.18 (m, 36H), 0.84 (t, 6H, *J* = 6.6 Hz) ppm. Anal. calcd for C_49_H_71_N_3_O_4_B_2_F_8_×H_2_O: C, 61.43; H, 7.69; N, 4.39; found: C, 61.31; H, 7.69; N, 4.44.

**1,1'-((3,5-Bis((dodecyloxy)carbonyl)-4-phenyl-1,4-dihydropyridine-2,6-diyl)bis(met­hylene))bis(4-methylpyridin-1-ium) dibromide (12)** was synthesized according to the literature [[3](#_ENREF_3)]:

**1,1'-((3,5-Bis((dodecyloxy)carbonyl)-4-phenyl-1,4-dihydropyridine-2,6-diyl)bis(methyle­ne))bis(4-(dimethylamino)pyridin-1-ium) dibromide (13)** was synthesized according to the literature [[3](#_ENREF_3)]:

**1,1'-((3,5-Bis((dodecyloxy)carbonyl)-4-phenyl-1,4-dihydropyridine-2,6-diyl)bis(met­hylene))bis(3-acetylpyridin-1-ium) dibromide (14)** was synthesized according to the literature [[3](#_ENREF_3)]:

**1,1'-((3,5-Bis((dodecyloxy)carbonyl)-4-phenyl-1,4-dihydropyridine-2,6-diyl)bis(methyl­ne))bis(1-methylpiperidin-1-ium) diperclorate (15)** was synthesized according to the literature [[3](#_ENREF_3)]:

**1,1'-((3,5-Bis((dodecyloxy)carbonyl)-4-phenyl-1,4-dihydropyridine-2,6-diyl)bis(methyl­ne))bis(pyrazin-1-ium) dibromide (16)** was synthesized according to the literature [[3](#_ENREF_3)]:

**N,N'-((3,5-Bis((dodecyloxy)carbonyl)-4-phenyl-1,4-dihydropyridine-2,6-diyl)bis(met­hylene))bis(N,N-dimethylcyclohexanaminium) diperchlorate (17)**

According to Scheme S1. 2,6-Di(bromomethyl)-3,5-bis(dodecyloxycarbonyl)-4-phenyl-1,4-dihydropyridine **2D** (1.0 eq) was dissolved in dry acetone after which 3.0 eq of corresponding amine was added. The reaction mixture was stirred at r.t. for 3 days, then solvent was evaporated and residue treated by concentrated (57%) HClO_4_ according to Turovska *et al.* [[9](#_ENREF_9)] to give comp **17**.

Yield: 43%, oil. ^1^H-NMR (CDCl_3_, 200 MHz, δ): 8.01 (s, 1H), 7.20 (s, 5H), 5.40 (m, 4H), 5.21 (s, 1H), 4.04 (s, 4H), 3.54**–**3.06 (m, 12H), 2.09**–**2.03 (m, 2H), 1.72**–**1.55 (m, 20H), 1.27 (br s, 40H), 0.88 (t, 6H) ppm. Anal. calcd for C_55_H_95_N_3_O_12_Cl_2_: C, 62.25; H, 9.02; N, 3.96; found: C, 62.01; H, 9.07; N, 3.95.

**1,1'-((3,5-Bis((dodecyloxy)carbonyl)-4-(4-hydroxyphenyl)-1,4-dihydropyridine-2,6-diyl)bis(methylene))bis(pyridin-1-ium) dibromide (18)**

According to Scheme S1. Corresponding 2,6-di(bromomethyl)-3,5-bis(dodecyloxycarbonyl)-4-(4-hydroxyphenyl)-1,4-dihydropyridine (**18D**) was obtained according to the procedure described by Rucins *et al.* [[4](#_ENREF_4)]. After then, 1.0 eq of 2,6-di(bromomethyl)-1,4-dihydropyridine **18D** was dissolved in acetone and treated by 2.1 eq of pyridine. Reaction mixture was stirred for 24 h at r.t, precipitate was filtered off and washed with acetone to give comp **18**.

Yield: 79%; Mp. 146-149°C decomp. ^1^H-NMR (DMSO-d_6_, 200 MHz, δ): 10.18 (s, 1H), 9.33 (s, 1H), 8.96 (d, 4H, *J* = 5.1 Hz), 8.57 (t, 2H, *J* = 6.7 Hz), 8.09 (dd, 4H, *J* = 6.7 and 5.1 Hz), 7.08 and 6.61 (2d, 4H, *J* = 8.1 Hz), 6.04 and 5.57 (AB-system, 4H, *J* = 14.6 Hz), 4.87 (s, 1H), 3.96 (t, 4H, *J* = 5.1 Hz), 1.56**–**1.42 (m, 4H), 1.29**–**1.16 (m, 36H), 0.83 (t, 6H, *J* = 5.9 Hz) ppm. Anal. calcd for C_49_H_71_N_3_O_5_Br_2_×2.5H_2_O: C, 59.63; H, 7.76; N, 4.26; found: C, 59.49; H, 7.33; N, 4.22.

**1,1'-((3,5-Bis((dodecyloxy)carbonyl)-4-(4-hydroxyphenyl)-1,4-dihydropyridine-2,6-diyl)bis(methylene))bis(4-methylpyridin-1-ium) dibromide (19)**

According to Scheme S1. Corresponding 2,6-di(bromomethyl)-3,5-bis(dodecyloxycarbonyl)-4-(4-hydroxyphenyl)-1,4-dihydropyridine (**18D**) was obtained according to the procedure described by Rucins *et al* [[4](#_ENREF_4)]. After then, 1.0 eq of 2,6-di(bromomethyl)-1,4-dihydropyridine **18D** was dissolved in acetone and treated by 2.1 eq of 4-methylpyridine. Reaction mixture was stirred for 24 h at r.t, precipitate was filtered off and washed with acetone to give comp **19**.

Yield: 56%; Mp. 147-148°C. ^1^H-NMR (DMSO-d_6_, 200 MHz, δ): 10.07 (br.s, 1H), 9.33 (s, 1H), 8.75 and 7.91 (2d, 8H, *J* = 6.6Hz), 7.03 and 6.61 (2d, 4H, *J* = 8.1 Hz), 5.93 and 5.47 (AB-system, 4H, *J* = 14.7 Hz), 4.86 (s, 1H), 3.97 (t, 4H, *J* = 5.9 Hz), 2.60 (s, 6H), 1.54**–**1.44 (m, 4H), 1.26**–**1.19 (m, 36H), 0.84 (t, 6H, *J* = 6.3 Hz) ppm. Anal. calcd for C_51_H_75_N_3_O_5_Br_2_×H_2_O: C, 62.00; H, 7.86; N, 4.25; found: C, 61.85; H, 7.91; N, 4.12.

**N,N'-((3,5-bis((dodecyloxy)carbonyl)-4-phenyl-1,4-dihydropyridine-2,6-diyl)bis(met­hylene))bis(N,N-dimethyldodecan-1-aminium) diperchlorate (20)**

Yield: 30%; Mp. (foam)°C. ^1^H-NMR (CDCl_3_, 400 MHz, δ): 8.01 (br s, 1H), 7.49**–**7.29 (m, 4H), 7.25**–**7.20 (m, 1H), 5.42 and 4.67 (two br s, 2×2H), 5.20 (s, 1H), 4.04 (t, 4H, *J* = 6.7 Hz), 3.49 and 3.15 (two br s, 2×6H), 3.38 (t, 4H, *J* = 8.2 Hz), 1.90**–**1.81 (m, 4H), 1.65**–**1.57 (m, 4H), 1.33**–**1.22 (m, 72H), 0.88 (t, 6H, *J* = 6.7 Hz), 0.87 (t, 6H, *J* = 7.0 Hz) ppm. ^13^C-NMR (CDCl_3_, 100 MHz, δ): 166.40, 145.25, 135.09, 129.50, 128.21, 127.91, 112.30, 77.20, 68.79, 65.43, 31.89, 31.87, 29.69, 29.63, 29.56, 29.53, 29.38, 29.34, 29.29, 29.26, 28.96, 28.40, 26.24, 25.98, 22.78, 22.65, 14.08 ppm. Anal. calcd for C_67_H_123_N_3_O_12_Cl_2_: C, 65.23; H, 10.05; N, 3.09; found: C, 64.92; H, 10.11; N, 3.09.

**1,1'-((3,5-Bis((dodecyloxy)carbonyl)-4-phenyl-1,4-dihydropyridine-2,6-diyl)bis(methyle­ne))bis(4-(trifluoromethyl)pyridin-1-ium) dibromide (21)** was synthesized according to the literature[[10](#_ENREF_10)]:

**1,1'-((4-phenyl-3,5-bis(((12,12,12-trifluorododecyl)oxy)carbonyl)-1,4-dihydropyridine-2,6-diyl)bis(methylene))bis(pyridin-1-ium) diperchlorate (22)** was synthesized according to the literature [[10](#_ENREF_10)]:

**Tetradodecyl 4,4'-(1,4-phenylene)bis(2,6-dimethyl-1,4-dihydropyridine-3,5-dicarbo­xylate) (26C)**

According to Scheme S1. The corresponding tetradodecyl 4,4'-(1,4-phenylene)bis(2,6-dimethyl-1,4-dihydropyridine-3,5-dicarboxylate) (**26C**) was obtained from dodecyl acetoacetate (**2B**) (4.0 eq), terephtalicaldehyde **26A** (1.0 eq) and ammonium acetate as a source of ammonia (2.4 eq) by classical Hantzsch synthesis.

Yield: 75%; Mp.136-137°C. ^1^H-NMR (CDCl_3_, 400 MHz, δ): 7.05 (s, 4H), 5.73 (br s, 2H), 4.95 (s, 2H), 4.01 and 4.00 (two t, 8H, *J* = 6.7 Hz), 2.29 (s, 12H), 1.59 (quint, 8H, *J* = 6.7 Hz), 1.33**–**1.24 (m, 72H), 0.88 (t, 12H, *J* = 6.7 Hz) ppm. ^13^C-NMR (CDCl_3_, 100 MHz, δ): 168.06, 145.27, 144.09, 127.62, 104.41, 64.17, 64.16, 39.03, 39.02, 32.22, 29.99, 29.97, 29.96, 29.88, 29.67, 29.58, 26.36, 22.97, 14.39 ppm. Anal. calcd for C_72_H_120_N_2_O_8_: C, 75.74; H, 10.59; N, 2.45; found: C, 75.79; H, 10.83; N, 2.45.

**Tetradodecyl 4,4'-(1,4-phenylene)bis(2,6-bis(bromomethyl)-1,4-dihydropyridine-3,5-dicarboxylate) (26D**)

Bromination of 2,6-methyl groups of 1,4-DHP **26C** was performed by N-bromosuccinimide (NBS) (4.0 eq) in methanol, the reaction mixture was stirred at r.t. for 12 h, after then a solvent was evaporated and the residue purified by flash chromatography, giving tetradodecyl 4,4'-(1,4-phenylene)bis(2,6-bis(bromomethyl)-1,4-dihydropyridine-3,5-dicarboxylate) (**26D**). Yield: 43%; oil, ^1^H-NMR (CDCl_3_, 400 MHz, δ): 7.07 (s, 4H), 6.45 (br s, 2H), 4.99 (s, 2H), 4.93 and 4.58 (AB-system, 8H, *J* = 11.3 Hz), 4.10**–**4.01 (m, 8H), 1.65**–**1.57 (m, 8H), 1.34**–**1.23 (m, 72H), 0.89**–**0.83 (m, 12H) ppm.

**1,1',1'',1'''-((1,4-Phenylenebis(3,5-bis((dodecyloxy)carbonyl)-1,4-dihydropyridine-4,2,6-triyl))tetrakis(methylene))tetrakis(pyridin-1-ium) tetrabromide** (**26**)

Tetradodecyl 4,4'-(1,4-phenylene)bis(2,6-bis(bromomethyl)-1,4-dihydropyridine-3,5-dicarboxylate) (**26D**) (1.0 eq) was dissolved in dry acetone after which dry pyridine (4.0 eq) was added, and the reaction mixture was stirred at r.t. overnight. The precipitate was filtered off and washed with acetone giving 1,1',1'',1'''-((1,4-phenylenebis(3,5-bis((dodecyl­oxy)carbonyl)-1,4-dihydropyridine-4,2,6-triyl))tetrakis(methylene))tetrakis(pyridin-1-ium) tetrabromide (**26**) as a pale yellow solid.

Yield: 53%; Mp. 185-187°C. ^1^H-NMR (DMSO-d_6_, 400 MHz, δ): 10.5 (br s, 2H), 8.99 (d, 8H, *J* = 6.1 Hz), 8.60 (t, 4H, *J* = 7.6 Hz), 8.09 (dd, 8H, *J* = 7.6 and 6.1 Hz), 7.23 (s, 4H), 6.21 and 5.58 (AB-system, 8H, *J* = 14.9 Hz), 4.99 (s, 2H), 4.05**–**3.90 (m, 8H), 1.55**–**1.51 (quint, 8H, *J* = 6.5 Hz); 1.36**–**1.22 (m, 72H), 0.85 (t, 12H, *J* = 6.5 Hz) ppm. ^13^C-NMR (DMSO-d_6_, 100 MHz, δ): 165.45, 146.20, 145.48, 144.48, 144.05, 138.49, 127.85, 127.55, 108.58, 64.44, 57.03, 31.29, 30.59, 29.20, 29.15, 29.12, 29.06, 28.78, 28.74, 27.86, 25.58, 22.03, 13.79 ppm. Anal. calcd for C_92_H_136_N_6_O_8_Br_4_×4H_2_O: C, 60.46; H, 7.83; N, 4.60; found: C, 60.36; H, 7.72; N, 4.34.

**1,1’-((3,5-Bis(dodecyloxycarbonyl)-4-phenylpyridine-2,6-diyl)bis(methylene))-bis(pyridin-1-ium)dibromide (27)** was synthesized according to the literature [[11](#_ENREF_11)]:

**Synthesis of compound 23**

**Scheme S2.** Synthesis of unsymmetrical 1,4-DHP amphiphile **23**.

DHP **23** was synthesized where the only one of two esters contains a perfluorinated ester moiety (Scheme S2). The enamine **23B** and benzylidene **23A** were reacted in diglyme at 80°C with n-butylpyridinium chloride as a PTC to yield the DHP **23C** as a pale yellow solid, which on bromination with NBS formed the 2,6-dibromomethyl DHP **23D** and without further purification was reacted with pyridine to provide the unsymmetrical 1,4-DHP **23** as a pale yellow solid having a broad Mp. 145-160°C.

**5,5,6,6,7,7,8,8,9,9,10,10,11,11,12,12,12-Heptadecafluorododecyl 2-benzylidene-3-oxobutanoate (*E/Z* mix) (23A)**

Compound **3B**, 2.88 g (0.005mol) and benzaldehyde (**1A**) 0.53 g (0.005mol) were dissolved in 40 mL benzene and 3 drops of glacial acetic acid and 5 drops of piperidine were added. The mixture was stirred at r.t. overnight. The solvent was evaporated and the residue was purified by silica gel chromatography (eluent: 10% EtOAc / hexane) providing 2.47 g of a yellow oil in 74% yield. ^1^H-NMR (CDCl_3_, 200 MHz, δ): 7.60 (s, 1H), 7.41 (m, 5H), 4.28 (t, 2H, *J* = 6.2 Hz), 2.44 (s, 3H), 2.02 (m, 2H), 1.79-1.49 (m, 4H) ppm.

**Dodecyl 5,5,6,6,7,7,8,8,9,9,10,10,11,11,12,12,12-heptadecafluorododecyl 1,4-dihydro-2,6-dimethyl-4-phenylpyridine-3,5-dicarboxylate (23C)**

1.88 g (0.003 mol) of compound **23B** and 0.75 g (0.003 mol) of compound **23A** were dissolved in diethyleneglycol 0.038 g 1-butylpyridinium chloride (11 mol%) was added and the flask was heated at 80°C for 5 h and after then stirring at r.t. overnight. Mixture poured into ice-water and extracted with EtOAc 3×40 mL. The organic layer was dried with Na_2_SO_4_, filtered and the solvent evaporated leaving an oily residue 2.63 g which was recrystallized from EtOH giving 1.10g of a pale yellow compound in 43% yield.

^1^H-NMR (CDCl3, 400 MHz, δ): 7.20–7.03 (m, 5H), 5.63 (br s, 1H), 4.91 (s, 1H), 4.07**–**3.91 (m, 4H), 2.28 (s, 3H), 2.26 (s, 3H), 2.03**–**1.89 (m, 2H), 1.86 (m, 18H), 1.62**–**1.49 (m, 6H), 0.81 (t, 3H, *J* = 6.2 Hz) ppm. ^13^C-NMR (CDCl_3_, 100 MHz, δ): 167.62, 167.45, 147.60, 144.46, 143.64, 127.89, 127.80, 126.20, 121.58-107.20 [(*C*F2)_7_*C*F_3_], 104.43, 103.74, 64.03, 62.80, 39.57, 30.45 (t, *J* = 21.8 Hz, *C*H2CF2), 19.59, 14.05 ppm.

**Dodecyl 5,5,6,6,7,7,8,8,9,9,10,10,11,11,12,12,12-heptadecafluorododecyl 2,6-bis(bromo­methyl)-4-phenyl-1,4-dihydropyridine-3,5-dicarboxylate (23D)**

0.60 g (0.0007 mol) of compound **23C** was dissolved in chloroform 6 mL and 4 mL MeOH and cooled in ice-bath then was added 0.24 g (0.0014 mol) of NBS. The mixture was stirred at r.t. overnight, then the solvent was removed under reduced pressure. The residue was triturated with carbontetrachloride and filtered to remove the precipitated succinimide. The solvent was evaporated to provide 0.52 g of a yellow compound in 69% yield.

^1^H-NMR (CDCl_3_, 200 MHz, δ): 7.26 (m, 5H), 6.93 (br s, 1H), 4,98 (s, 1H), 4.95**–**4.60 (dd, 4H, *J* = 11.6 and 56.4 Hz), 4.12 (m, 4H), 2.22-1.99 (m, 4H,), 1.62**–**1.52 (m, 6H), 1.86 (m, 18H), 0.81 (t, 3H, *J* = 6.2 Hz) ppm.

**Dodecyl 5,5,6,6,7,7,8,8,9,9,10,10,11,11,12,12,12-heptadecafluorododecyl 1,4-dihydro-2,6-dimethyl-4-phenylpyridine-3,5-dicarboxylate-2,6-dipyridinium dibromide (23)**

In 2 mL of dry acetone was dissolved 0.50 g (0.047 mmol) of compound **23D** and after adding 0.07 g (0.93 mmol) of dry pyridine mixture was stirred at r.t. overnight. The solid was filtered and washed with diethyl ether to give a pale yellow solid 0.28 g in 49% yield. Mp. 140-147°C. ^1^H-NMR (CDCl_3_, 400 MHz, δ): 10.92 (s, 1H), 10.18 (d, 4H, *J* = 9.2 Hz), 9.55 (t, 2H, *J* = 6.4 Hz), 9.30 (m, 4H), 7.22**–**7.16 (m, 5H), 6.12 (dd, *J* = 194 and 14.0 Hz, 4H), 5.03 (s, 1H), 4.06**–**3.98 (m, 4H), 1.98**–**1.95 (m, 4H), 1.63**–**1.51 (m, 6H), 1.86 (m, 18H), 0.81 (t, 3H, *J* = 6.2 Hz) ppm. LC/MS: MS(+ESI) m/z (rel.intensity): 1151 ([M-Br]^+^, 100).

**Synthesis of compound 24**

**5,5,6,6,7,7,8,8,9,9,10,10,11,11,12,12,12-Heptadecafluorododecyl 3-aminobut-2-enoate (23X)**

1.50g (0.0026mol) of compound **3B** was dissolved in 2 mL EtOH, then 3 mL of concentrated aqueous ammonia was added and the mixture stirred at r.t. overnight. The precipitated solid was filtered and washed with water. After drying there was obtained 0.94 g of a white powder in 63% yield. Mp. 75-76°C. ^1^H-NMR (CDCl_3_, 200 MHz, δ): 4.46 (s, 1H,), 4.02 (t, 2H, *J* = 6.0 Hz), 2.11**–**2.00 (m, 2H), 1.85 (s, 3H), 1.66**–**1.65 (m, 4H) ppm. LC/MS: MS(+ESI) m/z (rel.intensity): 598 ([M+Na]^+^, 100).

**Bis(5,5,6,6,7,7,8,8,9,9,10,10,11,11,12,12,12-heptadecafluorododecyl) 1,4-dihydro-2,6-dimethyl-4-phenylpyridine-3,5-dicarboxylate (24C)**

To a 1.33 g (0.002mol) of compound **23X** and 1.15 g (0.002 mol) of compound **3B** was added 11 mol % or 0.038 g of 1-butylpyridinium chloride then the mixture was dissolved in diethyleneglycol 10 mL and stirred at 80°C for 6 h then after stirred at r.t. overnight. The mixture was poured in ice-water and extracted with EtOAc 3×40 mL. The organic extract was dried with anh. Na_2_SO_4_, filtered and solvent was evaporated. The yellow residue (2.57 g) was recrystallized from EtOH to give 1.10 g of a pale yellow compound in 45% yield. Mp. 107-108°C ^1^H-NMR (CDCl_3_, 400 MHz, δ): 7.20**–**7.04 (m, 5H), 5.60 (br s, 1H), 4.89 (s, 1H), 4.06**–**3.95 (m, 4H), 2.28 (s, 6H), 2.04**–**1.91 (m, 4H), 1.63**–**1.52 (m, 8H) ppm. ^13^C-NMR (CDCl_3_, 100 MHz, δ): 167.34, 147.51, 144.31, 127.95, 127.81, 126.32, 120.86-107.30 [(*C*F2)_7_CF_3_], 103.99, 62.88, 39.54, 30.43 (t, *J* = 21.6 Hz, *C*H_2_CF_2_), 28.22, 19.54, 17.01 ppm.

**Di-5,5,6,6,7,7,8,8,9,9,10,10,11,11,12,12,12-heptadecafluorododecyl 2,6-bis(bromo­methyl)-4-phenyl-1,4-dihydropyridine-3,5-dicarboxylate (24D)**

0.62 g (0.0005 mol) of compound **24C** was dissolved in 6 mL of chloroform and 4 mL of MeOH. The solution was cooled at 0°C and 0.18 g (0.001 mol) of NBS was added in small portions then the reaction was stirred at r.t. overnight. The solvent was evaporated and the residue triturated with carbontetrachloride and filtered off. The solvent was removed in vacuum providing 0.53 g of a yellow solid in 75% yield. ^1^H-NMR (CDCl_3_, 200 MHz, δ): 7.26 (m, 5H), 6.93 (br s, 1H), 4.98 (s, 1H), 4.78 (dd, 4H, *J* = 11.6 and 56.4 Hz), 4.12 (m, 4H), 2.22**–**1.99 (m, 4H), 1.62**–**1.56 (m, 8H) ppm.

**Bis(5,5,6,6,7,7,8,8,9,9,10,10,11,11,12,12,12-heptadecafluorododecyl) 1,4-dihydro-2,6-dimethyl-4-phenylpyridine-3,5-dicarboxylate-2,6-dipyridinium bromide (24)**

0.45 g (0.33mmol) of compound **24D** was dissolved in dry acetone 2 mL then was added dry pyridine 0.05 g (0.66mmol) and stirred at r.t. overnight. The precipitates were filtered and washed with diethyl ether to provide 0.30 g of a pale yellow powder in 60% yield. Mp. 172-177°C. ^1^H-NMR (DMSO-d_6_, 400 MHz, δ): 10.30 (br s, 1H), 8.98 (d, 4H, *J* = 6.0 Hz), 8.59 (t, 2H, *J* = 8.4 Hz), 8.11 (m, 4H), 7.28**–**7.22 (m, 5H), 5.85 (dd, 4H*, J* = 100.8 and 15.2 Hz), 5.00 (s, 1H), 4.04 (m, 4H), 2.35**–**2.05 (m, 4H), 1.81**–**1.58 (m, 8H) ppm.

**Synthesis of compound 25**

**12,12,13,13,14,14,15,15,16,16,17,17,18,18,19,19,19-Heptadecafluorononadecyl 3-oxo­butanoate** (**4B**)

The mixture of 12,12,13,13,14,14,15,15,16,16,17,17,18,18,19,19,19-heptadecafluoronona­decyl alcohol (synthesized according to [[14](#_ENREF_14)]) and (2.0 g, 14 mmol) 2,2,6-trimethyl-4H-1,3-dioxin-4-one in 5 mL xylene was refluxed for 2 h. After the xylene was evaporated and the residue eluted through a silica gel column with dichloromethane there was obtained compound **4B** as a white solid Mp. 50-51°C in 96% yield. ^1^H-NMR (CDCl_3_, 200 MHz, δ): 4.13 (t, 2H, *J* = 7.0 Hz), 3.44 (s, 2H), 2.26 (s, 3H), 2.04 (tt, 2H, *J* = 8.2 and 18.9 Hz), 1.68**–**1.52 (m, 4H), 1.45**–**1.26 (m, 14H) ppm.

**Bis(12,12,13,13,14,14,15,15,16,16,17,17,18,18,19,19,19-heptadecafluorononadecyl) 1,4-dihydro-2,6-dimethyl-4-phenylpyridine-3,5-dicarboxylate (25C)**

Compound **4B** (1.5 g, 2 mmol), benzaldehyde (**1A**) (0.12 g, 1 mmol) and ammonium acetate (0.08 g, 1 mmol) in 5 mL of ethanol were weighted in a pressure tube and heated in a microwave oven at 120°C for 30 min. Reaction mixture was cooled and the precipitated yellowish solid filtered and washed with cold ethanol. The product was recrystallized from ethanol and ethyl acetate to give a light yellow solid 350 mg. Mp. 82-87°C in 25% yield. ^1^H-NMR (CDCl_3_, 200 MHz, δ): 7.28**–**7.10 (m, 5H), 5.63 (br. s, 1H), 4.99 (s, 1H), 4.01 (t, 4H, *J* = 6.5 Hz), 2.33 (s, 6H), 2.04 (tt, 4H, *J* = 8.2 and 18.9 Hz), 1.68**–**1.52 (m, 8H), 1.45**–**1.26 (m, 28H) ppm.

**Di-12,12,13,13,14,14,15,15,16,16,17,17,18,18,19,19,19-heptadecafluorononadecyl 2,6-bis(bromomethyl)-4-phenyl-1,4-dihydropyridine-3,5-dicarboxylate (25D)**

The compound **25C** (0.7 g, 0.5 mmol) was suspended in 20 mL of methanol and carbontetra­chloride was added until the solid dissolved then (0.18 g, 1 mmol) NBS was added and the solution was stirred at r.t. overnight. The solvent was evaporated and most of the solid dissolved in carbontetrachloride and the succinic acid was filtered off. The solvent was evaporated to yield a yellowish solid 0.60 g in 80% yield of **25D**. The compound was used in the next reaction without purification since it is unstable in solution. ^1^H-NMR (CDCl_3_, 200 MHz, δ): 7.28**–**7.10 (m, 5H), 6.46 (br. s, 1H), 5.01 (s, 1H), 4.92 and 4.61 (AB-system, 4H, *J* = 11.4 Hz), 4.01 (t, 4H, *J* = 4.0 and 6.5 Hz), 2.04 (tt, 4H, *J* = 8.2 and 18.9 Hz), 1.68**–**1.52 (m, 8H), 1.45**–**1.26 (m, 28H) ppm.

**Bis(12,12,13,13,14,14,15,15,16,16,17,17,18,18,19,19,19-heptadecafluorononadecyl)1,4-dihydro-2,6-dimethyl-4-phenylpyridine-3,5-dicarboxylate-2,6-dipyridinium dibromide (25)**

The 2,6-bis(bromomethyl)-1,4-dihydropyridine **25D** (0.19 g, 0.12 mmol) was dissolved in dry acetone and dry pyridine (0.025g, 0.3mmol) was added. The solution was stirred at r.t. overnight, precipitates was filtered to get a light yellow solid 0.14 g in 70% yield Mp. 85-105°C. ^1^H-NMR (CDCl_3_, 200 MHz, δ): 10.93 (br. s, 1H), 9.33 (d, 4H, *J* = 5.8 Hz), 8.57 (t, 2H, *J* = 7.8, 7.8 Hz), 8.18 (t, 4H, *J* = 7.6 and 6.6 Hz), 7.28**–**7.10 (m, 5H), 6.39 and 5.88 (AB-system, 4H, *J* = 13.8 Hz), 5.08 (s, 1H), 4.01 (t, 4H, *J* = 4.03, 6.46 Hz), 2.04 (tt, 4H, *J* = 8.2 and 18.9 Hz), 1.68**–**1.52 (m, 8H), 1.45**–**1.26 (m, 28H) ppm.

**Compounds from Table 2.**

**General procedure for synthesis of compounds 28–38**

Briefly, 3,4-dihydro-2(1*H*)-pyridone (DHPDO) (**C**) synthesis employed a four component reaction using Meldrum’s acid by a heterocyclization with a corresponding β-ketoester (**B**) and a corresponding aldehyde in the presence of ammonium acetate in refluxing glacial acetic acid [[12](#_ENREF_12)], (Scheme S3). After pouring the reaction mixture in ice-water the resulting solids were isolated by filtration and recrystallized from ethanol to provide white crystalline compounds. The DHPDO (**C**) was dissolved in chloroform and reacted with bromine giving the respective 6-methylbromides (**D**), which on subsequent reaction with pyridine or N,N-dimethyldodecyl-1-amine in dry acetone provided the DHPDO pyridinium or *N,N-*dimethyldodecan-1-aminium bromides **28–38.**

**Scheme S3.** Synthesis of 13,4-dihydro-2(1*H*)-pyridone (DHPDO) amphiphiles **28–38**.

**Compound 28**

**Methyl 4-[2-(difluoromethoxy)phenyl]-2-methyl-6-oxo-1,4,5,6-tetrahydropyridine-3-carboxylate (28C)**

Mp. 206-208°C. ^1^H-NMR (CDCl_3_, 400 MHz, δ): 8.03 (br s, 1H), 7.23 (m, 1H), 7.12 (m, 3H), 6.57 (t, 1H, *J* = 74.1 Hz), 4.63 (d, 1H, *J* = 8.4 Hz), 3.61 (s, 3H), 2.90 (dd, 1H, *J* = 8.5 and 16.6 Hz), 2.64 (ddd, 1H, *J* = 16.6, 1.9 and 1.0 Hz), 2.45 (s, 3H) ppm. ^13^C-NMR (CDCl_3_, 100 MHz, δ): 170.50, 167.02, 149.00, 147.42, 132.51, 128.48, 127.64, 125.61, 118.77, 116.41 (t, *J* = 258.2 Hz, CF_2_), 105.67, 51.44, 36.86, 31.94, 19.13 ppm.

**Methyl 2-(bromomethyl)-4-[2-(difluoromethoxy)phenyl]-6-oxo-1,4,5,6-tetrahydro­pyridine-3-carboxylate (28D)**

^1^H-NMR (CDCl_3_, 400 MHz, δ): 7.83 (br s, 1H), 7.25 (m, 1H), 7.15 (m, 3H), 6.58 (t, 1H, *J* =73.9 Hz), 5.04 (d, 1H, *J* = 11.1 Hz), 4.69 (dd, 1H, *J* = 8.4 and 1.6 Hz), 4.49 (d, 1H, *J* = 11.1 Hz), 3.65 (s, 3H), 2.95 (dd, 1H, *J* = 8.3 and 16.5 Hz), 2.68 (ddd, 1H, *J* = 16.8, 1.7 and 0.8 Hz) ppm. ^13^C-NMR (CDCl_3_, 100 MHz, δ): 169.44, 165.90, 148.86, 145.26, 131.61, 128.79, 127.33, 125.81, 118.70, 116.30 (t, *J* = 258.6 Hz, *C*F_2_), 108.42, 52.06, 36.64, 31.93, 26.15 ppm.

**1-({4-[2-(Difluoromethoxy)phenyl]-3-(methoxycarbonyl)-6-oxo-1,4,5,6-tetrahydro­pyridin-2-yl}methyl)pyridinium bromide (28)**

Mp. 181-183°C. ^1^H-NMR (DMSO-d_6_, 400 MHz, δ): 10.42 (s, 1H), 9.22–9.03 (m, 2H), 8.77–8.60 (m, 1H), 8.31–8.13 (m, 2H), 7.41–7.30 (m, 2H), 7.29–7.16 (m, 3H), 6.32 (d, *J* = 14.6 Hz, 1H), 5.50 (d, *J* = 14.7 Hz, 1H), 4.53 (dd, *J* = 8.2 and 2.0 Hz, 1H), 3.56 (s, 3H), 3.15 (dd, *J* = 16.2 and 8.2 Hz, 1H), 2.40–2.23 (m, 1H) ppm. ^13^C-NMR (CDCl3, 100 MHz, δ): 169.23, 166.06, 148.55, 146.48, 145.01, 142.24, 131.01, 128.81, 128.18, 127.65, 125.35, 119.34, 118.15, 116.78, 110.72, 58.12, 52.07, 36.36, 31.92 ppm. LC/MS: MS(+ESI) m/z (relative intensity): 389 ([M-Br]^+^ 100).

**Compound 29**

**Methyl 1-benzyl-2-methyl-6-oxo-4-phenyl-1,4,5,6-tetrahydropyridine-3-carboxylate (29C)** was synthesized according to the literature [[13](#_ENREF_13)]:

*Method A*: 0.51 g (0.20 mmol) of comp. **29C** was dissolved in dry CHCl_3_ and a Br_2_ solution in CHCl_3_ (0.20 mmol) was added drop-wise and stirred an additional 30 min. The CHCl_3_ was evaporated to give 0.77 g of an orange syrup. The syrup was passed through a silica gel plug with 10% EtOAc in petrol ether and after evaporation was obtained 0.31 g of a yellow oil in 50% yield.

*Method B*: 0.51 g (0.20 mmol) of comp. **29C** was dissolved in dry MeOH and while stirring NBS 0.36 g (0.20 mmol) was added after 1h stirred at r.t. the solvent was evaporated and the solids filtered and washed with CHCl_3_ and obtained a dark colored oil 0.46 g in 70% yield.

^1^H-NMR (CDCl_3_, 200 MHz, δ): 7.35**–**7.10 (m, 5H), 5.18 (s, 2H), 4.61 (s, 2H), 3.76 (s, 3H), 2.72**–**2.03 (m, 4H). LC/MS: MS(+ESI) m/z (relative intensity): 279 ([M+K]^+^ 100).

**1-{[1-Benzyl-3-(methoxycarbonyl)-6-oxo-1,4,5,6-tetrahydropyridin-2-yl]methyl}pyridi­nium perchlorate (29)**

0.38 g (0.0011 mol) of comp. **29D** was dissolved in dry acetone. Pyridine 0.09 g (0.0011 mol) was added and the reaction mixture was stirred at r.t. overnight. Then solvent was removed to give a brown syrup which was dissolved in abs. EtOH and conc. HClO_4_ was added by drops. The solvent was removed and after addition of diethethyl ether the oil solidified to give a pink solid 0.41 g in 77% yield.

^1^H-NMR (CDCl_3_, 400 MHz, δ): 8.56 (d, 2H, *J* = 4.0 Hz), 8.22**–**8.18 (m, 1H), 7.73**–**7.70 (m, 2H), 7.07**–**6.93 (m, 5H), 5.92 (s, 2H), 5.12 (s, 2H), 3.84 (s, 3H), 2.91**–**2.80 (m, 4H) ppm. ^13^C-NMR (CDCl_3_, 100 MHz, δ): 168.73, 164.80, 143.27, 141.65, 138.38, 134.27, 127.15, 126.09, 125.61, 123.99, 117.72, 55.53, 50.86, 43.09, 28.23, 19.43 ppm. LC/MS: MS(+ESI) m/z (relative intensity): 382 ([M-Br]^+^ 100).

**(3-(((5,5,6,6,7,7,8,8,9,9,10,10,11,11,12,12,12-Heptadecafluorododecyl)oxy)carbonyl)-6-oxo-4-phenyl-1,4,5,6-tetrahydropyridin-2-yl)triphenylphosphonium bromide (30)** in accordance was synthesized according to the literature [[14](#_ENREF_14)]:

**1-((3-(((5,5,6,6,7,7,8,8,9,9,10,10,11,11,12,12,12-Heptadecafluorododecyl)oxy)carbonyl)-6-oxo-4-phenyl-1,4,5,6-tetrahydropyridin-2-yl)methyl)pyridin-1-ium bromide (31)** was synthesized according to the literature [[14](#_ENREF_14)]:

**1-((3-(((5,5,6,6,7,7,8,8,9,9,10,10,11,11,12,12,12-Heptadecafluorododecyl)oxy)carbonyl)-6-oxo-4-phenyl-1,4,5,6-tetrahydropyridin-2-yl)methyl)pyridin-1-ium perchlorate (32)**

The ClO_4_ salt was obtained by dissolving 20mg (0.023mmol) of **31**in 5mL EtOH and adding conc. HClO_4_ dropwise until the salt precipitated. The white solid was filtered and washed with Et_2_O and dried, affording 20mg of compound **32** in 98% yield. Mp. 179-180℃. ^1^H-NMR (CDCl_3_, 400 MHz, δ): 9.27 (s, 1H), 9.10–8.96 (m, 2H), 8.47 (tt, *J* = 7.7 and 1.3 Hz, 1H), 8.14–7.98 (m, 2H), 7.36–7.20 (m, 5H), 7.20–7.07 (m, 2H), 6.29 (d, *J* = 14.5 Hz, 1H), 5.57 (d, *J* = 14.5 Hz, 1H), 4.29 (dd, *J* = 8.5 and 2.5 Hz, 1H), 4.19–4.00 (m, 2H), 3.19 (dd, *J* = 17.2 and 8.6 Hz, 1H), 2.77 (dd, *J* = 17.1 and 2.6 Hz, 1H), 1.94 (tt, *J* = 18.3 and 8.0 Hz, 2H), 1.68–1.46 (m, 2H), 1.39 (p, *J* = 7.9 Hz, 2H) ppm. ^13^C-NMR (CDCl_3_, 100 MHz, δ): 166.50, 160.96, 160.53, 160.10, 159.67, 146.46, 145.05, 140.10, 138.78, 129.51, 128.90, 128.11, 126.44, 118.67, 116.35, 115.84, 113.01, 110.17, 65.33, 59.10, 38.41, 37.40, 27.83, 16.92 ppm. LC/MS: MS(+ESI) m/z (relative intensity): 783 ([M-ClO_4_]^+^ 100).

***N-*((3-(methyloxycarbonyl)-6-oxo-4-phenyl-1,4,5,6-tetrahydropyridin-2-yl)methyl-*****N,N-*dimethyldodecan-1-aminium bromide (33)**

Following the general synthesis there was obtained 79% yield of a white solid. C_28_H_45_BrN_2_O_3_ Mp. 186-187°C. ^1^H-NMR (CDCl_3_, 400 MHz, δ): 10.18 (br s, 1H), 7.29**–**7.12 (m, 5H), 5.46 (d, 1H, *J* = 12.0 Hz), 5.15 (d, 1H, *J* = 12.0 Hz), 4.28 (m, 1H), 3.62 (s, 3H), 3.60**–**3.34 (m, 8H), 2.58**–**2.54 (m, 2H), 1.36**–**1.27 (m, 20H), 0.89 (t, 6H, *J* = 8.0 Hz) ppm. ^13^C-NMR (CDCl_3_, 100 MHz, δ): 167.08, 164.73, 138.83, 136.99, 127.08, 125.32, 124.73, 114.42, 66.33, 56.49, 50.28, 36.50, 29.97, 27.66, 27.65, 27.52, 27.39, 20.75, 12.19 ppm. LC/MS: MS(+ESI) m/z (rel.intensity): 457 ([M-Br]^+^, 100).

***N*-((3-(dodecyloxycarbonyl)-6-oxo-4-phenyl-1,4,5,6-tetrahydropyridin-2-yl)methyl-*N,N*-dimethyldodecan-1-aminium bromide (34)**

0.25 g (0.52 mmol) of 5-methoxycarbonyl-6-bromomethyl-4-phenyl-3,4-dihydro-2(1H)-pyridone was dissolved in dry acetone (10 mL). While stirring 0.12 g (0.52 mmol) of N,N-dimethyldodecyl-1-amine was added and the mixture was stirred at r.t. overnight and the precipitated solid was filtered and washed with diethyl ether to give 0.28 g of a white solid in 78% yield. Mp 152-154°C ^1^H-NMR (CDCl_3_, 200 MHz, δ): 10.26 (br s, 1H), 7.27**–**7.09 (m, 5H), 5.47 (d, 1H, *J* = 12.0 Hz), 5.20 (d, 1H, *J* = 12.0 Hz), 4.20 (d, 1H, *J =* 5.8 Hz), 4.15 (t, 2H, *J* = 6.0 Hz), 3.58**–**3.46 (m, 2H), 3.41 (s, 6H), 2.82**–**2.55 (m, 2H), 1.52**–**1.40 (m, 2H) 1.34**–**1.25 (m, 20H), 1.24**–**1.15 (m, 18H), 0.87 (t, 6H, *J* = 6.0 Hz) ppm. LC/MS: MS(+ESI) m/z (rel. intensity): 612 ([M-Br]^+^, 100).

***N*-((3-((5,5,6,6,7,7,8,8,9,9,10,10,11,11,12,12,12-Heptadecafluorododecyloxy)carbonyl)-6-oxo-4-phenyl-1,4,5,6-tetrahydropyridin-2-yl)methyl-*N,N*-dimethyldodecan-1-aminium bromide (35)**

Using the general synthesis there was obtained a white solid in 63% yield. C_39_H_50_BrF_17_N_2_O_3_ Mp. 173-174°C. ^1^H-NMR (CDCl_3_, 400 MHz, δ): 10.26 (br s, 1H), 7.27**–**7.09 (m, 5H), 5.47 (d, 1H, *J* = 12.0 Hz), 5.20 (d, 1H, *J* = 12.0 Hz), 4.20 (d, 1H, *J =* 5.8 Hz), 4.15 (t, 2H, *J* = 6.0 Hz), 3.58**–**3.46 (m, 2H), 3.41 (s, 6H), 2.82**–**2.55 (m, 2H), 2.25**–**1.80 (m, 2H), 1.78**–**1.69 (m, 4H), 1.34**–**1.25 (m, 20H), 0.87 (t, 3H, *J* = 6.0 Hz) ppm. LC-MS: MS(+ESI) m/z (relative intensity): 918 ([M-Br]^+^ 100).

**N-((3-(methyloxycarbonyl)-6-oxo-1,4,5,6-tetrahydropyridin-2-yl)methyl-N,N-dimethyl­dodecan-1-aminium bromide (36)**

Following the general synthesis there was obtained a white solid in 79% yield.

^1^H-NMR (CDCl_3_, 400 MHz, δ): 10.08 (br s, 1H), 5.15 (s, 2H), 3.75 (s, 3H), 3.57**–**3.53 (m, 2H), 3.45 (s, 6H), 2.76 (br s, 2H), 2.67 (br s, 2H), 1.89 (br s, 2H), 1.38**–**1.27 (m, 20H), 0.89 (t, 6H, *J* = 8.0 Hz) ppm. ^13^C-NMR (CDCl_3_, 100 MHz, δ): 168.21, 164.81, 136.34, 111.96, 66.15, 56.94, 50.23, 29.97, 27.66, 27.64, 27.52, 27.48, 27.39, 27.22, 24.46, 21.03, 20.74, 12.18 ppm. LC/MS: MS(+ESI) m/z (relative intensity): 382 ([M-Br]^+^ 100).

***N*-((3-(dodecyloxycarbonyl)-6-oxo-1,4,5,6-tetrahydropyridin-2-yl)methyl-*N,N*-dimethyl­dodecan-1-aminium bromide (37)**

Following the general procedure there was obtained a white solid in 87% yield. Mp. 157-158°C. ^1^H-NMR (CDCl_3_, 200 MHz, δ): 10.26 (br s, 1H), 5.18 (br s, 2H), 4.15 (t, 2H, *J*=6 Hz), 3.58**–**3.46 (m, 2H), 3.41 (s, 6H), 2.82**–**2.55 (m, 2H), 1.52**–**1.40 (m, 2H) 1.34**–**1.25 (m, 20H), 1.24**–**1.15 (m, 18H), 0.87 (t, 6H, *J* = 6.0 Hz). LC/MS: MS(+ESI) m/z (relative intensity): 536 ([M-Br]^+^ 100).

***N*-((3-((5,5,6,6,7,7,8,8,9,9,10,10,11,11,12,12,12-Heptadecafluorododecyloxy)carbonyl)-6-oxo-1,4,5,6-tetrahydropyridin-2-yl)methyl-*N,N*-dimethyldodecan-1-aminium bromide (38)**

Following the general procedure there was obtained a white solid in 76% yield.

Mp.158-159°C. ^1^H-NMR (CDCl_3_, 200 MHz, δ): 10.17 (br s, 1H), 5.18 (br s, 2H), 4.15 (t, 2H, *J* = 6.0 Hz), 3.58**–**3.46 (m, 2H), 3.41 (s, 6H), 2.82**–**2.55 (m, 4H), 2.25**–**1.80 (m, 2H), 1.78**–**1.69 (m, 4H), 1.34**–**1.25 (m, 20H), 0.87 (t, 3H, *J* = 6.0 Hz). LC/MS: MS(+ESI) m/z (relative intensity): 842 ([M-Br]^+^ 100)

**Compounds from Table 3.**

**Scheme S4**. Synthesis of 2-amino-3-alkoxycarbonylalkylammonium halides **39–48**.

Previously (2-amino-3-alkoxycarbonylalkyl)trialkylammonium halides were synthesized and described as ribonucleic acid transfection agents.[[15](#_ENREF_15)]

Briefly, the first step included the transesterification of commercially available ethyl 4-chloroacetoacetate (**A**) with corresponding carbinol at 150-155°C without solvent with azeotropic removal of ethanol. The further reaction of corresponding 4-chloro-3-oxobutanates **B** with ammonium acetate in ethanol at 60°C leaded to enaminoesters **C**, which were used for quaternization of tertiary amines or heterocycles by long heating in dry acetone or methyl ethyl ketone. Potassium iodide or sodium bromide additives were used for obtaining corresponding iodides or bromides.

**General procedure for the synthesis of alkyl 4-chloro-3-oxobutanates B.**

The mixture of ethyl 4-chloro-3-oxobutanoate (1.0 eq) and corresponding alcohol (1.12 eq) was heated with stirring at 150-155°C for 7 h with azeotropic removal of ethanol. After cooling the crude products were filtered and crystallized from methanol, yielding target compounds, which were kept in refrigerator.

**Decyl 4-chloro-3-oxobutanoate (1B)**

The title compound was synthesized according to the general procedure from ethyl 4-chloro-3-oxobutanoate and decanol to afford target compound (93%) as yellowish crystalls. Mp<65°C. ^1^H-NMR (CDCl_3_, 400 MHz, δ): 4.11 (s, 2H), 3.11 (t, 2H, *J* = 6.7 Hz), 2.61 (s, 2H), 1.53**–**1.50 (m, 2H), 1.24**–**1.19 (m, 14H), 0.82 (t, 3H, *J* = 6.7 Hz) ppm.

**Dodecyl 4-chloro-3-oxobutanoate (2B)**

The title compound was synthesized according to the general procedure from ethyl 4-chloro-3-oxobutanoate and dodecanol to afford target compound (92%) as light brown crystalls. Mp<65°C. ^1^H-NMR (CDCl_3_, 400 MHz, δ): 4.11 (s, 2H), 3.11 (t, 2H, *J* = 6.7 Hz), 2.61 (s, 2H), 1.53**–**1.50 (m, 2H), 1.24**–**1.19 (m, 14H), 0.82 (t, 3H, *J* = 6.7 Hz) ppm.

**Hexadecyl 4-chloro-3-oxobutanoate (3B)**

The title compound was synthesized according to the general procedure from ethyl 4-chloro-3-oxobutanoate and hexadecanol to afford target compound (92%) as yellowish crystalls. Mp<65°C. ^1^H-NMR (CDCl_3_, 400 MHz, δ): 4.16 (s, 2H), 4.09 (t, 2H, *J* = 6.7 Hz), 3.15 (s, 2H), 1.60**–**1.55 (m, 2H), 1.22**–**1.19 (m, 26H), 0.82 (t, 3H, *J =* 6.5 Hz) ppm.

**General procedure for the synthesis of alkyl (*Z,E*)-3-amino-4-chlorobut-2-enoates (C)**

The mixture of corresponding 4-chloro-3-oxobutanate **B** (1.0 eq) with ammonium acetate (1.1 eq) was heated in ethanol at 60°C for 6 h. After cooling the crude products were filtered and crystallized from methanol, yielding target compounds.

**Decyl (*Z,E*)-3-amino-4-chlorobut-2-enoate (1C)**

Yield: 85 %, yellowish crystalls. Mp<65°C. ^1^H-NMR (DMSO-d_6_, 400 MHz, δ): 4.56 (s, 1H), 4.08 (s, 2H), 3.96 (t, 2H, *J* = 6.7 Hz), 1.49**–**1.41 (m, 2H), 1.20**–**1.16 (m, 14H), 0.83 (t, 3H, *J =* 6.7 Hz) ppm.

**Dodecyl (*Z,E*)-3-amino-4-chlorobut-2-enoate (2C)**

Yield: 79%, light brown crystals, Mp<65°C. ^1^H-NMR (DMSO-d_6_, 400 MHz, δ): 4.63 (s, 1H), 4.05 (s, 2H), 3.97 (t, 2H, *J* = 6.7 Hz), 1.52**–**1.46 (m, 2H), 1.31**–**1.26 (m, 18H), 0.88 (t, 3H, *J =* 6.8 Hz) ppm.

**Hexadecyl (*Z,E*)-3-amino-4-chlorobut-2-enoate (3C)**

Yield: 74%, light brown crystals, Mp<65°C. ^1^H-NMR (DMSO-d_6_, 400 MHz, δ): 4.56 (s, 1H), 4.07 (s, 2H), 3.89 (t, 2H, *J* = 6.7 Hz), 1.59**–**1.54 (m, 2H), 1.22**–**1.18 (m, 26H), 0.79 (t, 3H, *J =* 6.7 Hz) ppm.

**General procedure for the synthesis of (*Z,E*) amino-3-alkoxycarbonylalkylammonium halides 39–48**

To the mixture of corresponding alkyl (*Z,E*)-3-amino-4-chlorobut-2-enoate **C** (1.0 eq) in dry acetone or methylethylketone corresponding tertiary amine or heterocycle (2.1 eq) was added, after which the reaction mixture was heated at 75°C for 20 h. Potassium iodide or sodium bromide (2.0 eq) additives were used for obtaining corresponding iodides or bromides.

After cooling the crude products were filtered. Solvent was evaporated in vacuo, after which the residue was treated with dry dichloromethane, filtered. The solvent was evaporated, residue was treated with dry acetone, crystals were filtered off and crystallized from methanol, yielding the target compounds.

**(*Z,E*)-N-(2-Amino-4-(hexadecyloxy)-4-oxobut-2-en-1-yl)-N,N-dimethylcyclohexan­aminium chloride (39)**

Yield: 23%; white solid. M.p. 170-173°C. ^1^H-NMR (CDCl_3_, 400 MHz, δ): 8.13**–**8.05 (br.s, 2H), 4.54 (s, 2H), 4.51 (s, 1H), 4.06 (t, 2H, *J =* 6.8 Hz), 3.73**–**3.67 (m, 1H), 3.26 (s, 6H), 2.33**–**2.04 (m, 4H), 1.79**–**1.76 (m, 2H), 1.69**–**1.58 (m, 2H), 1.55**–**1.42 (m, 4H), 1.30**–**1.25 (m, 26H), 0.87 (t, 3H, *J =* 6.8 Hz) ppm. UPLC-ESI (m/z): 452 [M-Cl]^+^. Anal. calcd. for C_28_H_55_ClN_2_O_2_: C, 69.02; H, 11.40; N, 5.75. Found: C, 68.66; H, 11.66; N, 5.54.

**(*Z,E*)-1-(2-Amino-4-(hexadecyloxy)-4-oxobut-2-en-1-yl)-1-methylpyrrolidin-1-ium chloride (40)**

Yield: 44%; white solid. M.p. 118-120°C. ^1^H-NMR (CDCl_3_, 400 MHz, δ): 7.86 (s, 2H), 4.69 (s, 2H), 4.63 (s, 1H), 4.06 (t, 2H, *J =* 6.8 Hz), 4.02**–**3.97 (m, 2H), 3.71**–**3.66 (m, 2H), 3.33 (s, 3H), 2.39**–**2.35 (m, 2H), 2.23**–**2.17 (m, 2H), 1.65**–**1.58 (m, 2H), 1.35**–**1.25 (m, 26H), 0.86 (t, 3H, *J =* 6.8 Hz) ppm. ^13^C-NMR (CDCl_3_, 100 MHz, δ): 169.1, 149.9, 89.9, 65.2, 64.4, 63.6, 48.6, 31.9, 29.6, 25.9, 22.6, 21.1, 14.1 ppm. UPLC-ESI (m/z): 410 [M-Cl]^+^. Anal. calcd. for C_25_H_49_ClN_2_O_2_: C, 67.45; H, 11.12; N, 6.29. Found: C, 67.32; H, 11.36; N, 6.18.

**(*Z,E*)-2-Amino-N-(2-(dimethylamino)ethyl)-4-(hexadecyloxy)-N,N-dimethyl-4-oxobut-2-en-1-aminium chloride (41)**

Yield: 41%; white solid. M.p. 93-95°C. ^1^H-NMR (CDCl_3_, 400 MHz, δ): 7.87 (s, 2H), 4.67 (s, 2H), 4.63 (s, 1H), 4.06 (t, 2H, *J =* 6.8 Hz), 3.80 (t, 2H, *J =* 5.4 Hz), 3.44 (s, 6H), 2.82 (t, 2H, *J* = 5.4 Hz), 2.30 (s, 6H), 1.65-1.58 (m, 2H), 1.35**–**1.20 (m, 26H), 0.87 (t, 3H, *J* = 6.8 Hz) ppm. ^13^C-NMR (CDCl_3_, 100 MHz, δ): 169.1, 149.2, 90.5, 67.3, 63.6, 62.1, 53.9, 51.5, 45.2, 31.9, 29.6, 25.9, 22.7, 14.1 ppm. UPLC-ESI (m/z): 440 [M-Cl]^+^. Anal. calcd. for C_26_H_54_ClN_3_O_2_: C, 65.57; H, 11.45; N, 8.83. Found: C, 65.65; H, 11.71; N, 8.53.

**(*Z,E*)-1-(2-Amino-4-(hexadecyloxy)-4-oxobut-2-en-1-yl)-1,4-dimethylpiperazin-1-ium chloride (42)**

Yield: 33%; white solid. M.p. 135-138°C. ^1^H-NMR (CDCl_3_, 400 MHz, δ): 7.93 (s, 2H), 4.72 (s, 2H), 4.54 (s, 1H), 4.06 (t, 2H, *J* = 6.8 Hz), 3.88**–**3.66 (m, 4H), 3.49 (s, 3H), 2.94-2.82 (m, 2H), 2.74**–**2.60 (m, 2H), 2.40 (s, 3H), 1.65-1.58 (m, 2H), 1.35**–**1.20 (m, 26H), 0.88 (t, 3H, *J* = 6.8 Hz) ppm. ^13^C-NMR (CDCl_3_, 100 MHz, δ): 168.9, 148.5, 90.6, 63.6, 60.6, 48.4, 45.1, 31.9, 29.6, 29.3, 28.8, 25.9, 22.6, 14.1 ppm. UPLC-ESI (m/z): 438 [M-Cl]^+^. Anal. calcd. for C_26_H_52_ClN_3_O_2_: C, 65.85; H, 11.08; N, 8.86. Found: C, 65.94; H, 11.31; N, 8.59.

**(*Z,E*)-N-(2-Amino-4-(hexadecyloxy)-4-oxobut-2-en-1-yl)-N,N-dimethylhexan-1-aminium chloride (43)**

Yield: 37%; white solid. M.p. 178-180°C. ^1^H-NMR (CDCl_3_, 400 MHz, δ): 7.92 (s, 2H), 4.59 (s, 2H), 4.54 (s, 1H), 4.05 (t, 2H, *J* = 6.8 Hz), 3.52 (t, 2H, *J* = 8.5 Hz), 3.33 (s, 6H), 1.79**–**1.72 (m, 2H), 1.63**–**1.60 (m, 2H), 1.25**–**1.23 (m, 32H), 0.87 (t, 6H, *J* = 6.8 Hz) ppm. UPLC-ESI (m/z): 454 [M-Cl]^+^. Anal. calcd. for C_28_H_57_ClN_2_O_2_: C, 68.74; H, 11.77; N, 5.73. Found: C, 68.37; H, 11.98; N, 5.51.

**(*Z,E*)-N-(2-Amino-4-(hexadecyloxy)-4-oxobut-2-en-1-yl)-N,N-dimethyldodecan-1-aminium bromide (44)**

Yield: 26%; white solid. M.p. 73-74°C. ^1^H-NMR (CDCl_3_, 400 MHz, δ): 7.81**–**7.52 (m, 2H), 4.64 (s, 2H), 4.55 (s, 1H), 4.07 (t, 2H, *J* = 6.8 Hz), 3.57**–**3.53 (m, 2H), 3.35 (s, 6H), 1.81**–**1.74 (m, 2H), 1.66**–**1.58 (m, 2H), 1.30**–**1.21 (m, 44H), 0.88 (t, 6H, *J* = 6.8 Hz) ppm. UPLC-ESI (m/z): 537 [M-Br]^+^. Anal. calcd. for C_34_H_69_BrN_2_O_2_: C, 66.19; H, 11.29; N, 4.54. Found: C, 66.17; H, 11.46; N, 4.53.

**(*Z,E*)-N-(2-Amino-4-(hexadecyloxy)-4-oxobut-2-en-1-yl)-N,N-dimethyldodecan-1-aminium chloride (45)**

Yield: 33%, light brown solid. M.p. 165-168°C. ^1^H-NMR (CDCl_3_, 400 MHz, δ): 7.92 (s, 2H), 4.61(s, 2H), 4.56 (s, 1H), 4.06 (t, 2H, *J* = 6.9 Hz), 3.56**–**3.52 (m, 2H), 3.34 (s, 6H), 1.79**–**1.73 (m, 2H), 1.66**–**1.58 (m, 2H); 1.36**–**1.25 (m, 44H), 0.89**–**0.86 (m, 6H) ppm. UPLC-ESI (m/z): 537 [M-Cl]^+^. Anal. calcd. for C_34_H_69_ClN_2_O_2_: C, 71.22; H, 12.15; N, 4.88. Found: C, 71.08; H, 12.37; N, 4.67.

***(Z,E*)-N-(2-Amino-4-(hexadecyloxy)-4-oxobut-2-en-1-yl)-N,N-dimethyldodecan-1-aminium iodide (46)**

Yield 33%; light brown solid; M.p. 73-75°C. ^1^H-NMR (CDCl_3_, 400 MHz, δ): 8.02**–**7.78 (m, 2H), 4.68 (s, 2H), 4.64 (s, 1H), 4.07 (t, 2H, *J* = 6.9 Hz), 3.63**–**3.58 (m, 2H), 3.37 (s, 6H), 1.80**–**1.75 (m, 2H), 1.66-1.58 (m, 2H), 1.38**–**1.25 (m, 44H), 0.88 (t, 6H, *J =* 6.9 Hz) ppm. UPLC-ESI (m/z): 537 [M-I]^+^. Anal. calcd. for C_34_H_69_IN_2_O_2_: C, 61.42; H, 10.48; N, 4.21. Found: C, 61.46; H, 10.46; N, 3.92.

**(*Z,E*)-1-(2-Amino-4-(dodecyloxy)-4-oxobut-2-en-1-yl)pyridin-1-ium iodide (47)**

Yield: 53%; light brown solid. M.p. 97-99°C. ^1^H-NMR (CDCl_3_, 400 MHz, δ): 9.45 (dd, 2H, *J =* 6.7 and 1.5 Hz), 8.53 (tt, 1H,  *J* = 7.8 and 1.5 Hz), 8.10 (dd, 2H, *J =* 7.8 and 6.7 Hz), 7.21**–**7.16 (br.s, 2H), 5.75 (s, 2H), 4.82 (s, 1H), 4.06 (t, 2H, *J =* 6.8 Hz), 1.63-1.55 (m, 2H), 1.32**–**1.25 (m, 18H), 0.88 (t, 3H, *J =* 6.8 Hz) ppm.

**(*Z,E*)-N-(2-Amino-4-(decyloxy)-4-oxobut-2-en-1-yl)-N,N-dimethyldodecan-1-aminium chloride (48)**

Yield: 80%; light yellow solid. M.p. 74-76°C. ^1^H-NMR (CDCl_3_, 400 MHz, δ): 7.91 (s, 2H), 4.62 (s, 2H), 4.57 (s, 1H), 4.06 (t, 2H, *J* = 6.9 Hz), 3.56**–**3.52 (m, 2H), 3.35 (s, 6H), 1.82**–**1.72 (m, 2H), 1.64**–**1.60 (m, 2H), 1.45**–**1.26 (m, 32H), 0.88 (t, 6H, *J* = 6.9 Hz) ppm. Anal. calcd. for: C_28_H_57_ClN_2_O_2_: C, 68.74; H, 11.74; N, 5.73. Found: C, 68.61; H, 12.64; N, 5.65.

**References:**

1. Hyvonen, Z., et al., *Novel cationic amphiphilic 1,4-dihydropyridine derivatives for DNA delivery.* Biochim Biophys Acta, 2000. **1509**(1-2): p. 451-66.

2. Pajuste, K., et al., *Use of pyridinium ionic liquids as catalysts for the synthesis of 3,5-bis(dodecyloxycarbonyl)-1,4-dihydropyridine derivative.* Central European Journal of Chemistry, 2011. **9**(1): p. 143-148.

3. Pajuste, K., et al., *Gene delivery agents possessing antiradical activity: Self-assembling cationic amphiphilic 1,4-dihydropyridine derivatives.* New Journal of Chemistry, 2013. **37**(10): p. 3062-3075.

4. Rucins, M., et al., *Data for the synthesis and characterisation of 2,6-di(bromomethyl)-3,5-bis(alkoxycarbonyl)-4-aryl-1,4-dihydropyridines as important intermediates for synthesis of amphiphilic 1,4-dihydropyridines.* Data in Brief, 2020. **30**: p. 105532.

5. Plotniece, A., et al., *Oxidation of cationic 1,4-dihydropyridine derivatives as model compounds for putative gene delivery agents.* Tetrahedron, 2009. **65**(40): p. 8344-8349.

6. Pajuste, K., et al., T*he synthesis of new cationic 1,4-dihydropyridine derivatives for DNA delivery*. Rigas Tehniskas Universitates Zinatniskie Raksti, Serija 1: Materialzinatne un Lietiska Kimija, 2005, **11**: p. 7-10.

7. Petrova, M., et al., *Intramolecular C-H⋯O hydrogen bonding in 1,4-dihydropyridine derivatives.* Molecules, 2011. **16**(9): p. 8041-8052.

8. Bruvere, I., et al., *Dihydropyridine derivatives as cell growth modulators in vitro.* Oxidative Medicine and Cellular Longevity, 2017. **2017**.

9. Turovska, B., et al., *Electrochemical oxidation of compounds containing 1,4-dihydropyridine and pyridinium rings - Analogs of gene transfection agents.* Chemistry of Heterocyclic Compounds, 2004. **40**(6): p. 753-758.

10. Muhamadejev, R., et al., *Study of interactions of mononucleotides with 1,4-dihydropyridine vesicles using NMR and ITC techniques.* New Journal of Chemistry, 2018. **42**(9): p. 6942-6948.

11. Petrichenko, O., et al., *Studies of the physicochemical and structural properties of self-assembling cationic pyridine derivatives as gene delivery agents.* Chemistry and Physics of Lipids, 2015. **191**: p. 25-37.

12. Morales, A., et al., *Novel hexahydrofuro[3,4-b]-2(1H)-pyridones from 4-aryl substituted 5-alkoxycarbonyl-6-methyl-3,4-dihydropyridones.* Journal of Heterocyclic Chemistry, 1996. **33**(1): p. 103-107.

13. Smits, R., et al., *Synthesis of 5-carboxy-6-methyl-3,4-dihydro-2(1H)-pyridone derivatives and their electrochemical oxidation to 2-pyridones.* Chemical Physics Letters, 2016. **649**: p. 84-87.

14. Smits, R., et al., *Synthesis and self-assembly of novel fluorous cationic amphiphiles with a 3,4-dihydro-2(1H)-pyridone spacer.* Journal of Fluorine Chemistry, 2011. **132**(6): p. 414-419.

15. Timofejeva, I., Kozlovska, T., Vezane, A., Apsite, G., Ose-Klinklava, V., Bruvere, R., Vigante, B., Plotniece, A., Bisenieks, E., Duburs, G. *Novel biologically active enaminoester derivatives as agent for chemotherapy*. LV patent P-16-111, **20.07.2018**.
